# Supplementary material for: Dynamics of Immune Cell Infiltration and Fibroblast-Derived IL-33/ST2 Axis Induction in a Mouse Model of Post-Surgical Lymphedema
Source: Int J Mol Sci. 2025 Feb 6;26(3):1371. doi: 10.3390/ijms26031371 (PMC11818732; doi:10.3390/ijms26031371)
Supplement: Supplementary file 1 [file ijms-26-01371-s001.zip › Supplementary_Material.pdf]

Supplementary Material

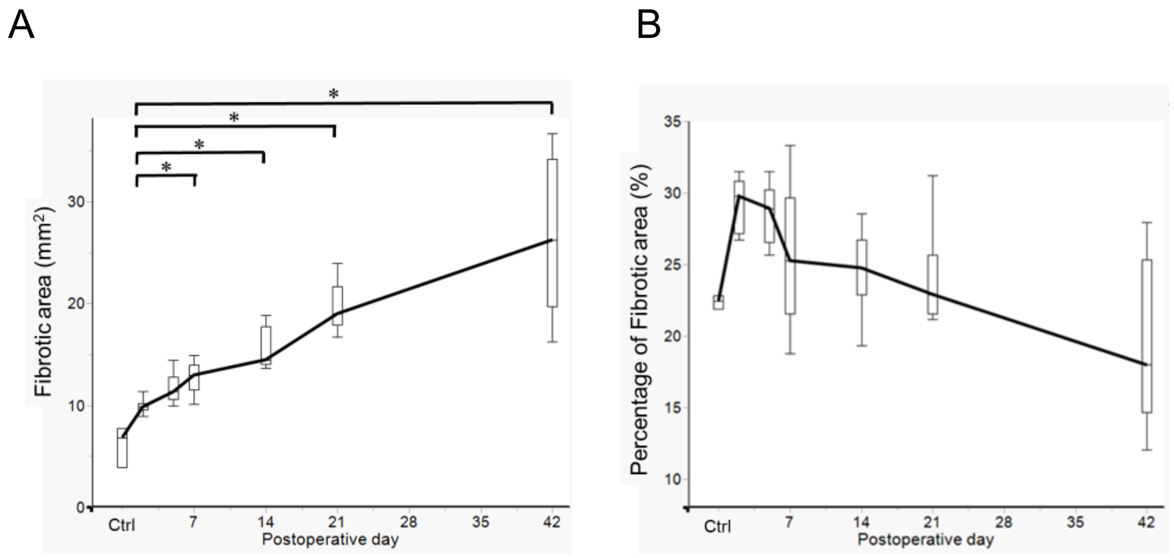

Figure S1. Dermal and subcutaneous fibrosis of the tail lymphedema tissue in non-operated controls (Ctrl) and on postoperative days (POD) 2, 5, 7, 14, 21, and 42.

A. Variation in fibrotic area (mm^2) in dermal and subcutaneous lymphedema tissues (Ctrl: n = 3; POD 2, 5, 7, 14, and 21: n = 8; POD 42: n = 6). Boxes represent 50 % of the data, with medians (lines), interquartile ranges (whiskers). The Steel–Dwass test was used to determine the time points that were significantly different from POD 2. *$P < 0.05$.

B. Variation in the percentage of fibrotic area (%) in dermal and subcutaneous lymphedema tissue (Ctrl: n = 3; POD 2, 5, 7, 14, and 21: n = 8; POD 42: n = 6). Boxes represent 50 % of the data, with medians (lines), interquartile ranges (whiskers). The Steel-Dwass test revealed no significant differences between the time points.

Ctrl

CD4 DAPI

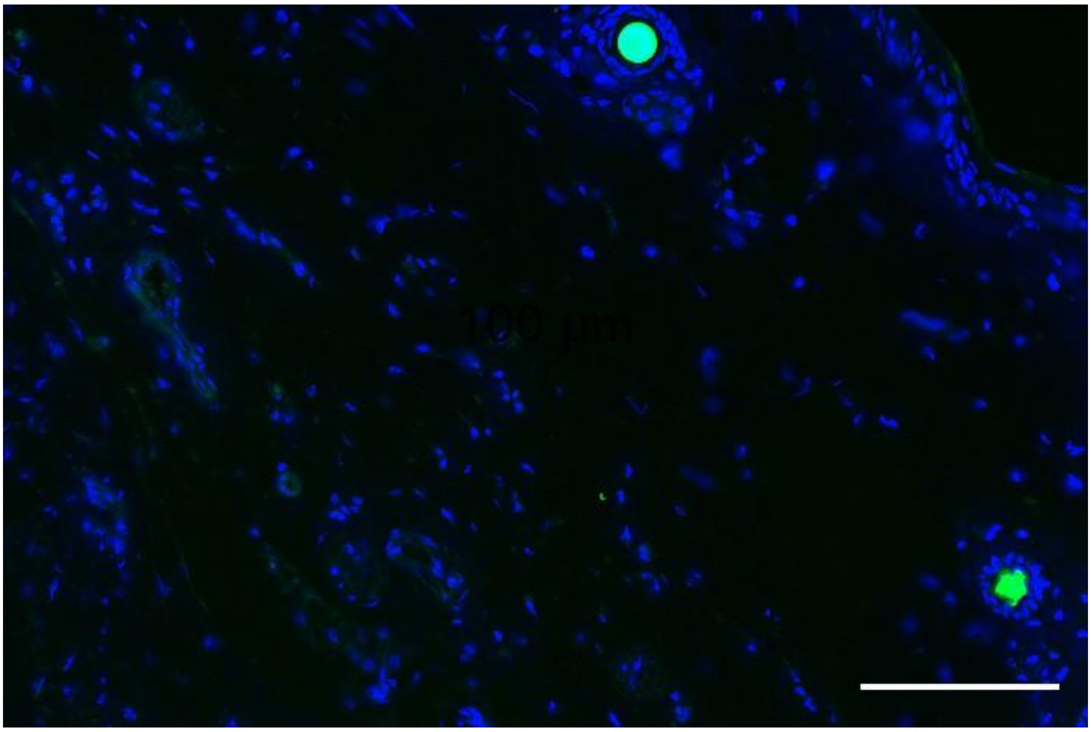

POD 2

CD4 DAPI

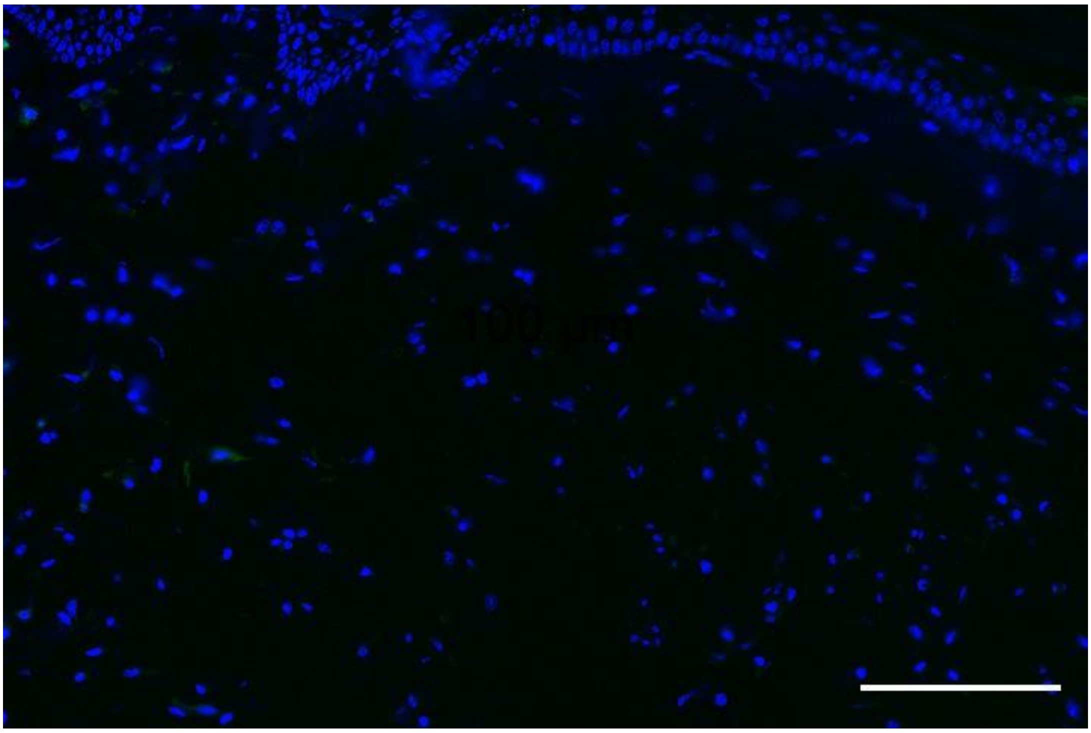

POD 5

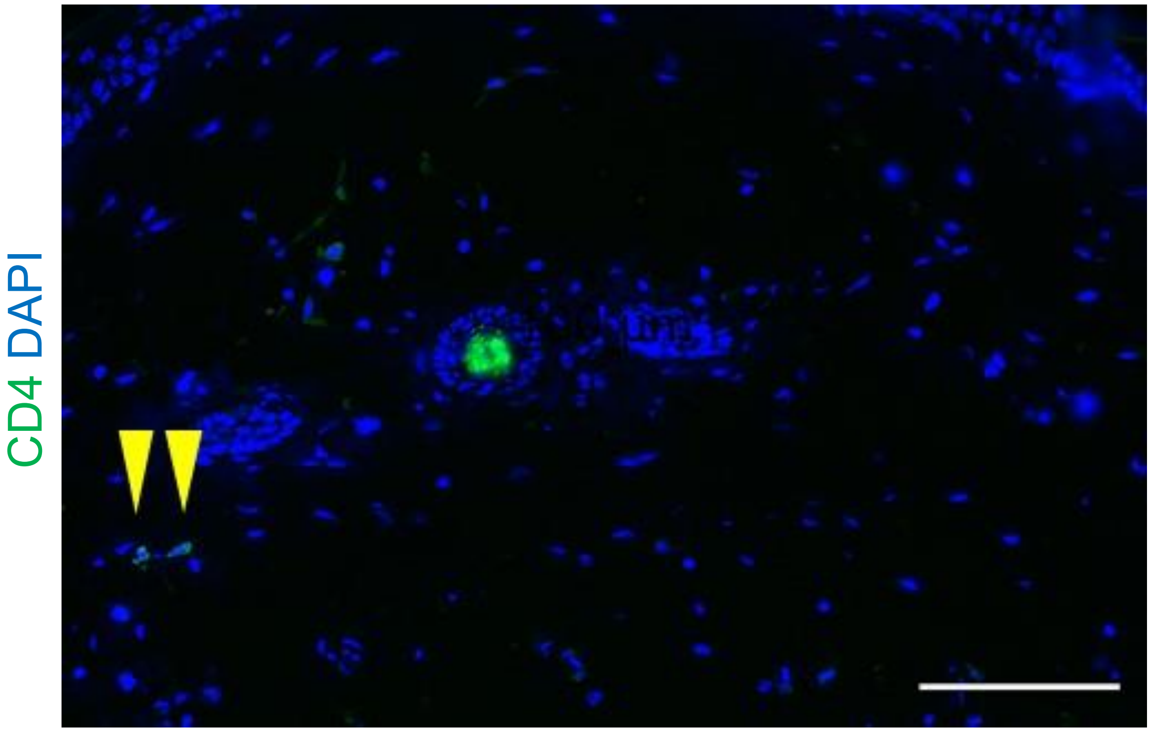

POD 7

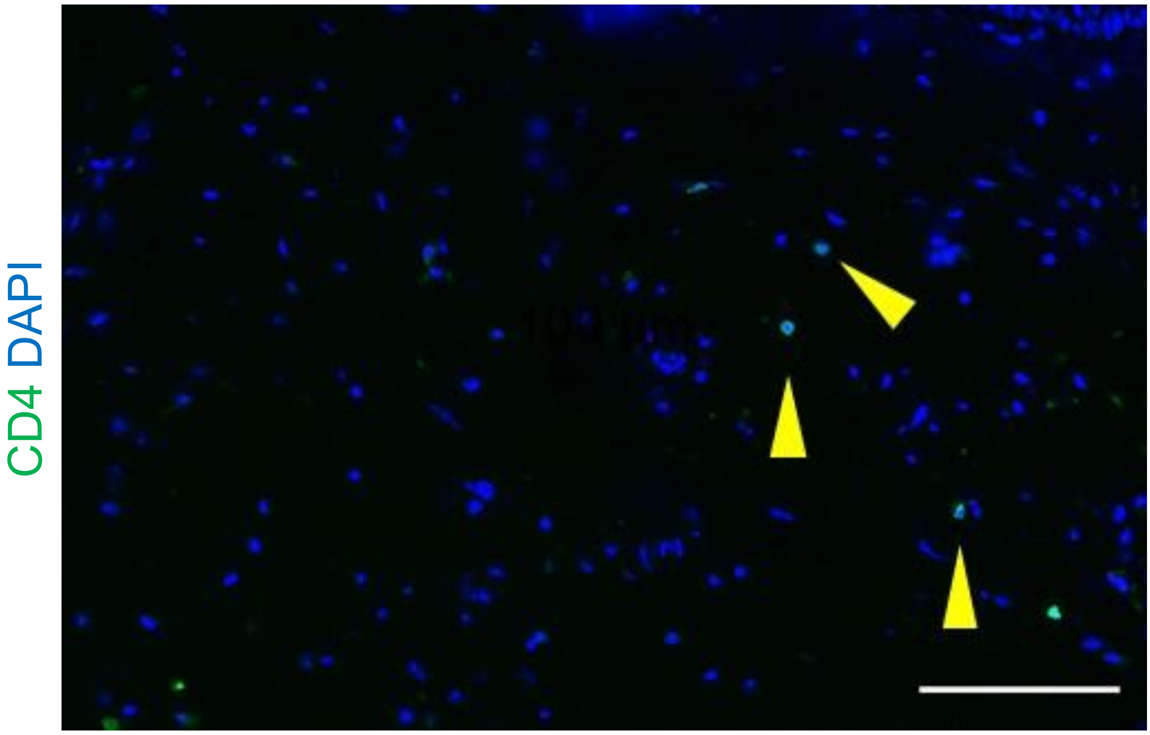

POD 14

CD4 DAPI

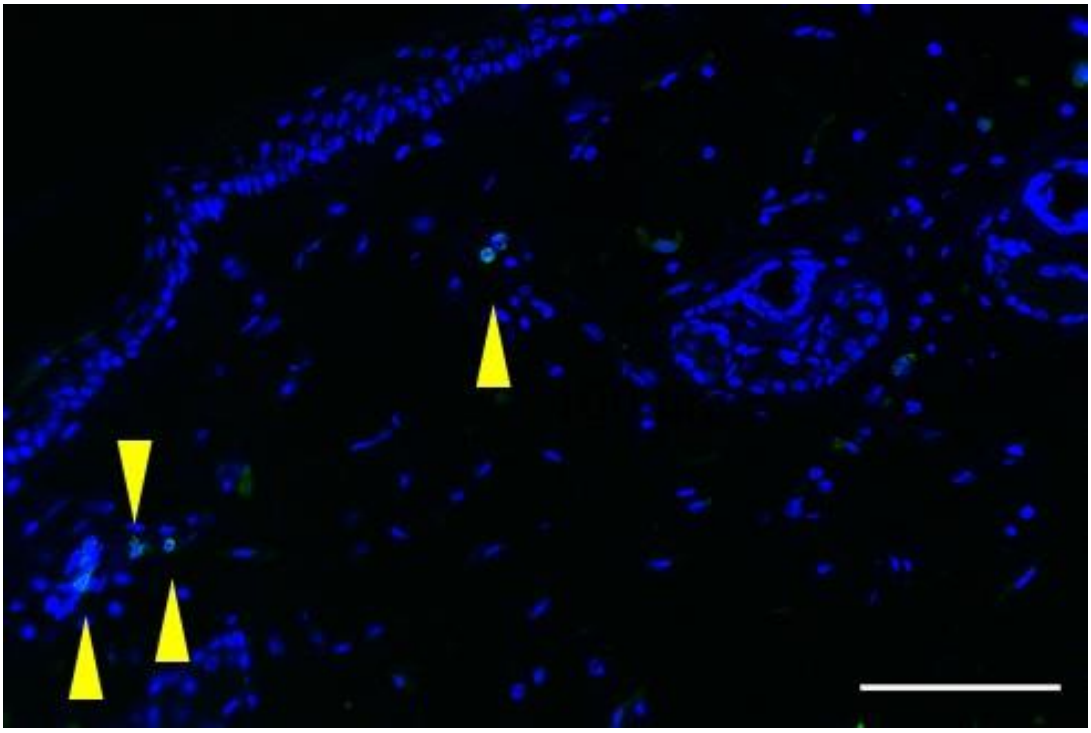

POD 21

CD4 DAPI

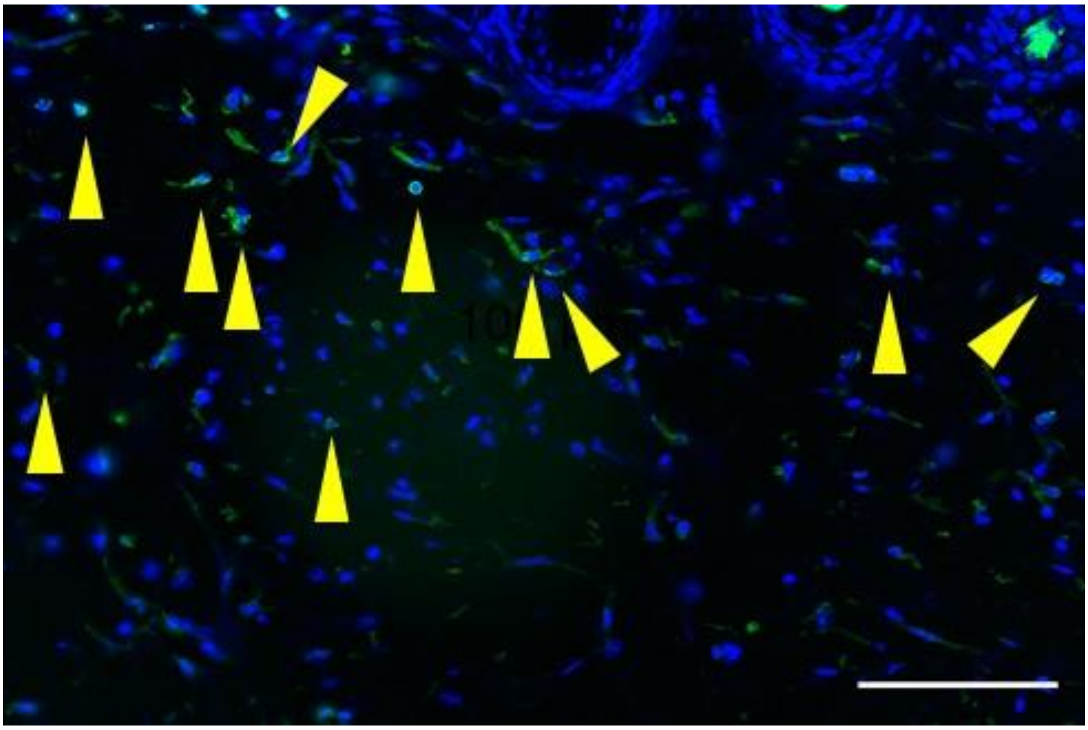

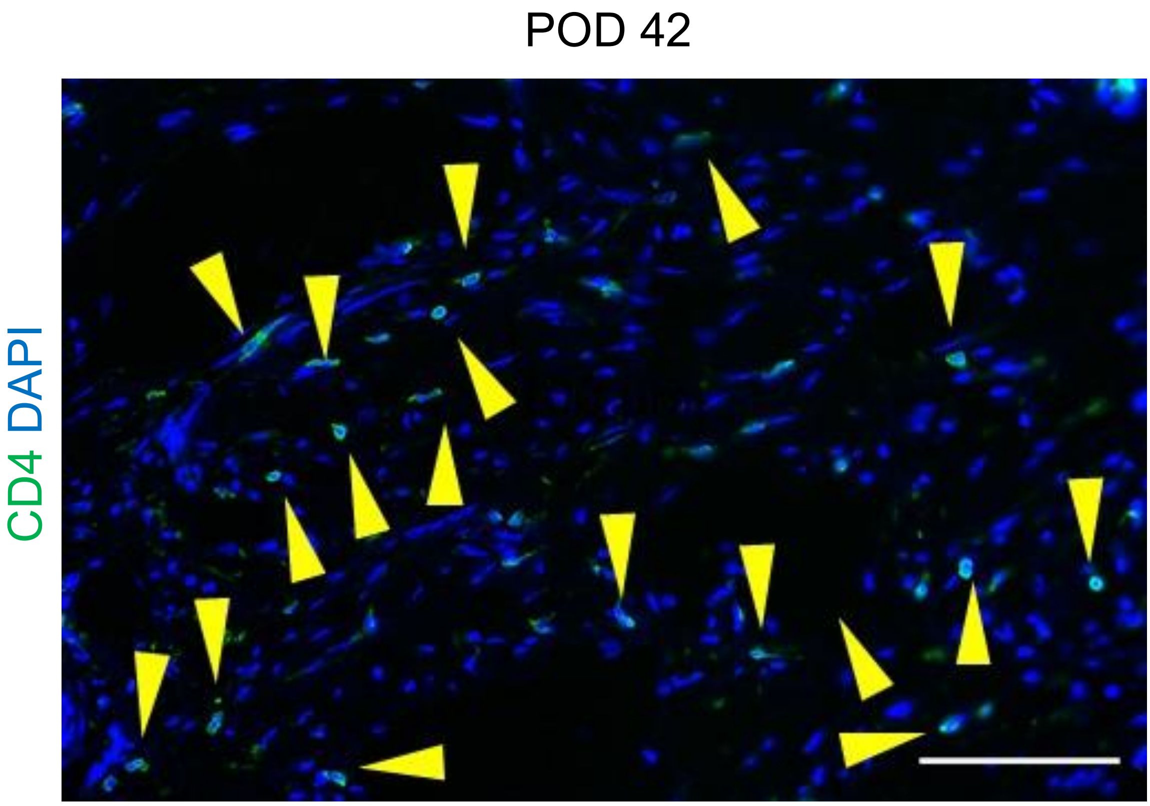

Figure S2. Enlarged views of Fig. 2A.

POD 14

CD4 LYVE-1 DAPI

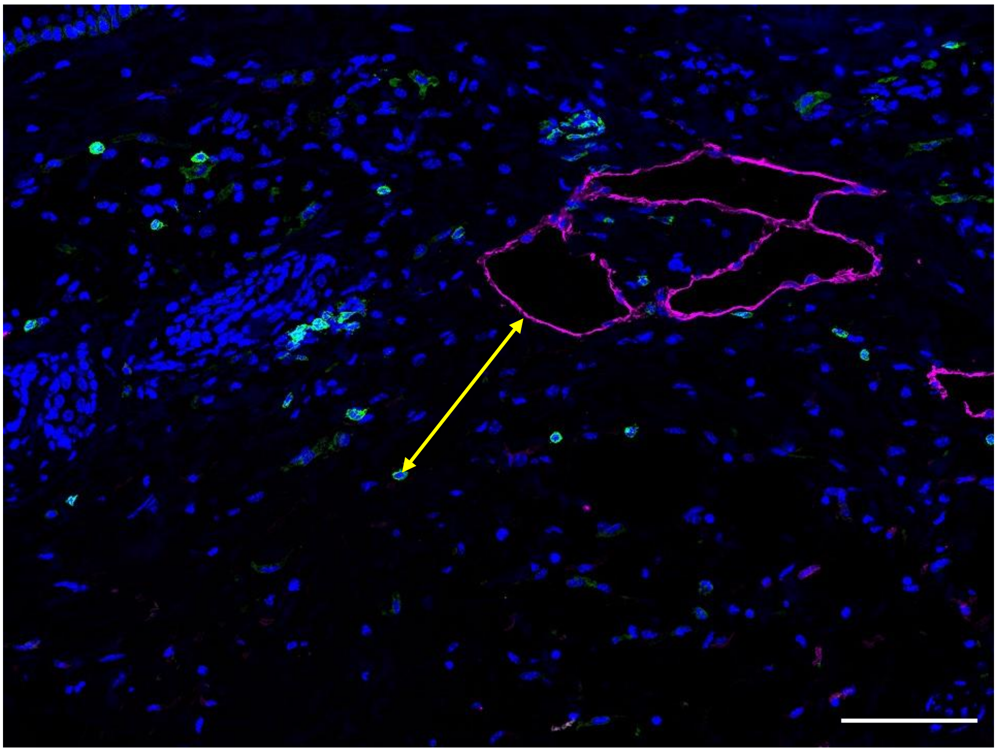

POD 21

CD4 LYVE-1 DAPI

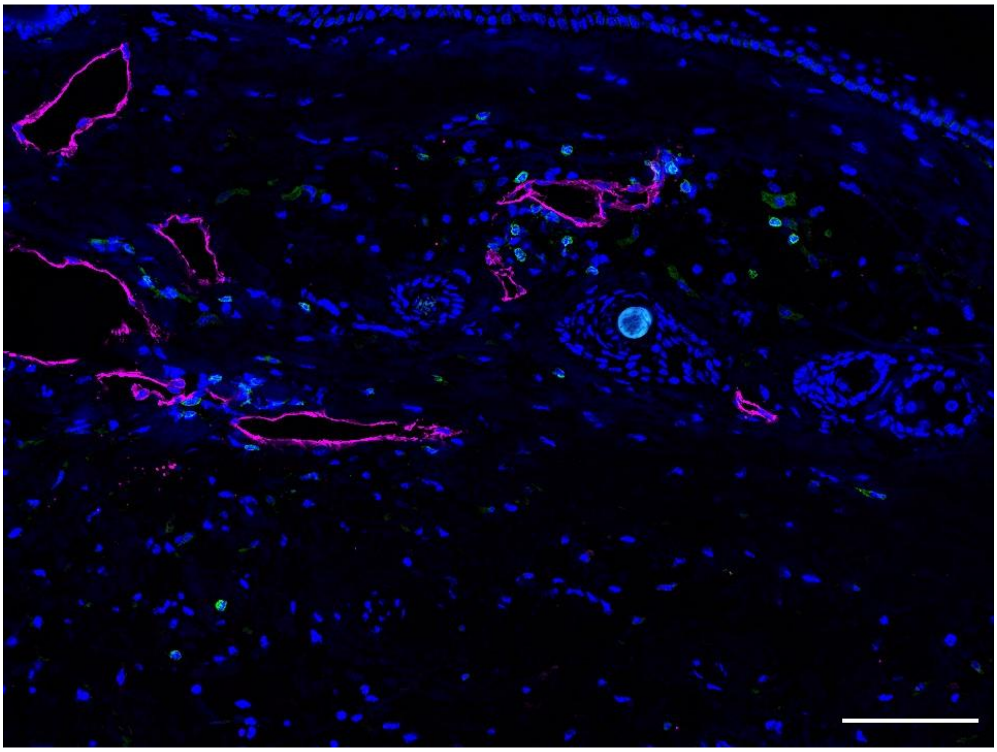

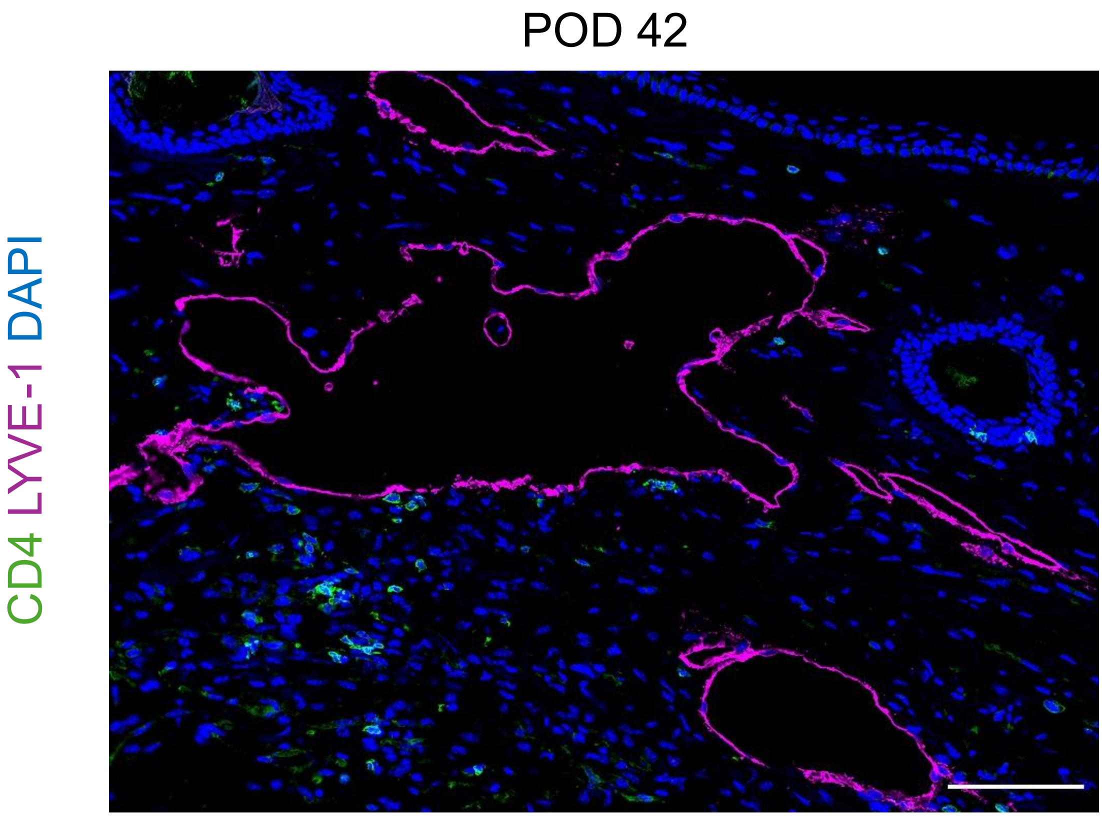

Figure S3. Representative images of the distance between CD4^{+} cells (green) and lymphatic vessels(magenta) on postoperative days (POD) 14, 21, and 42. The yellow double-headed arrow on POD 14 indicates the representative distance between the CD4^{+} cells (green) and lymphatic vessels (magenta). Nuclei are stained with 4′,6-diamidino-2-phenylindole (DAPI) (blue). Scale bar = 100 μm

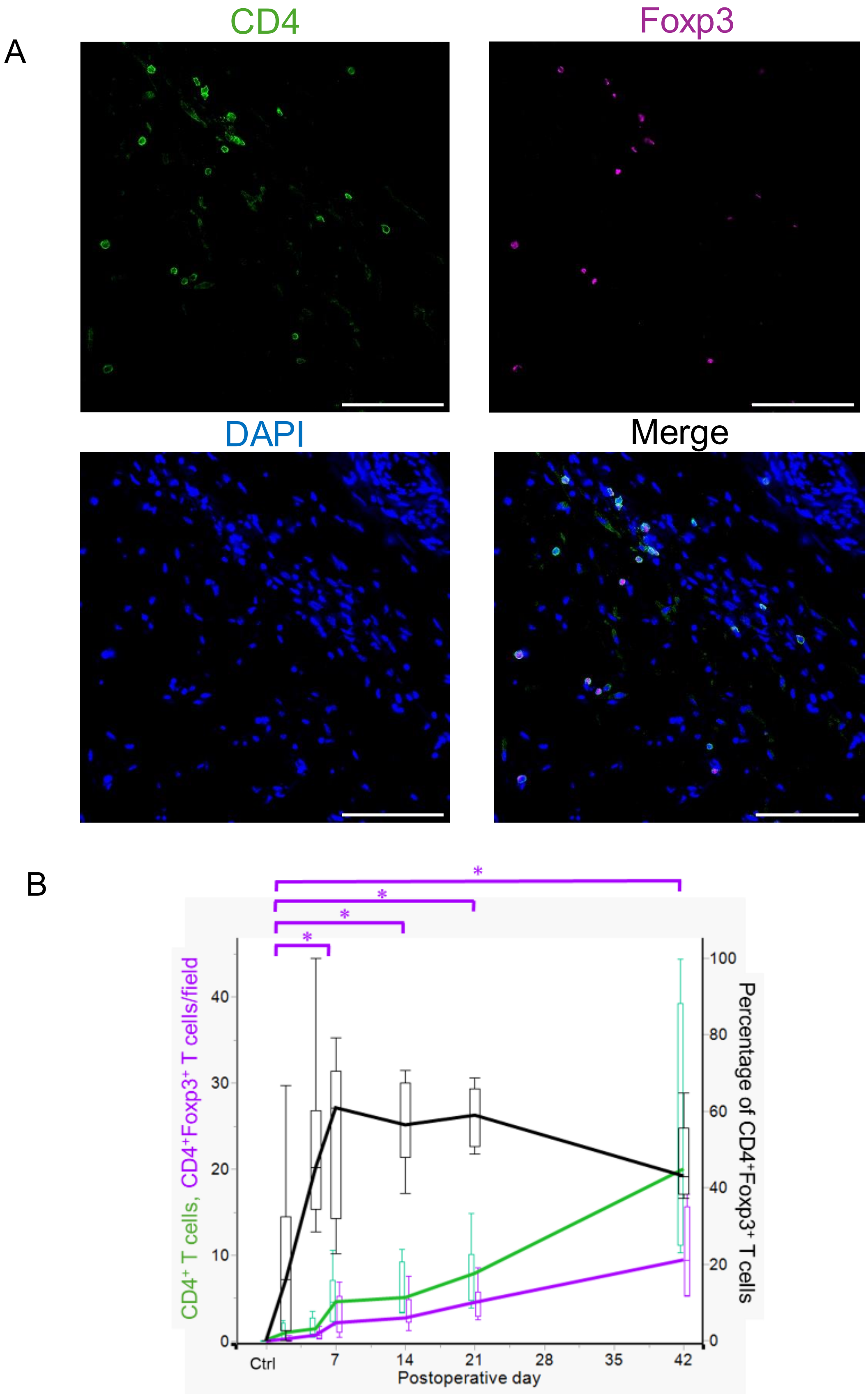

Figure S4. Infiltration of CD4$^+$FOXP3$^+$ regulatory T cells (Tregs) in the tail lymphedema tissue of non-operated controls (Ctrl) and on postoperative days (POD) 2, 5, 7, 14, 21, and 42

A. Representative images of CD4$^+$ T cells (green) and Foxp3$^+$ cells (magenta) in the lymphedema tissues (POD14). Nuclei are stained with 4′,6-diamidino-2-phenylindole (DAPI) (blue). Scale bar = 100 μm

B. Variation in the numbers of $CD4^+$ T cells (green) and $CD4^+FOXP3^+$ T cells (magenta) per field (8 fields/mouse) infiltrating in the lymphedema tissue (Ctrl: n = 3; POD 2, 5, 7, 14, and 21: n = 8; POD 42: n = 6). The percentage of $CD4^+Foxp3^+$ T cells among $CD4^+$ T cells is indicated by a black line. Boxes represent 50 % of the data, with medians (lines), interquartile ranges (whiskers). The Steel–Dwass test was used to determine the time points that were significantly different from POD 2 in terms of the number (indicated by magenta bars) and percentage of $CD4^+Foxp3^+$ T cells. *$P < 0.05$.

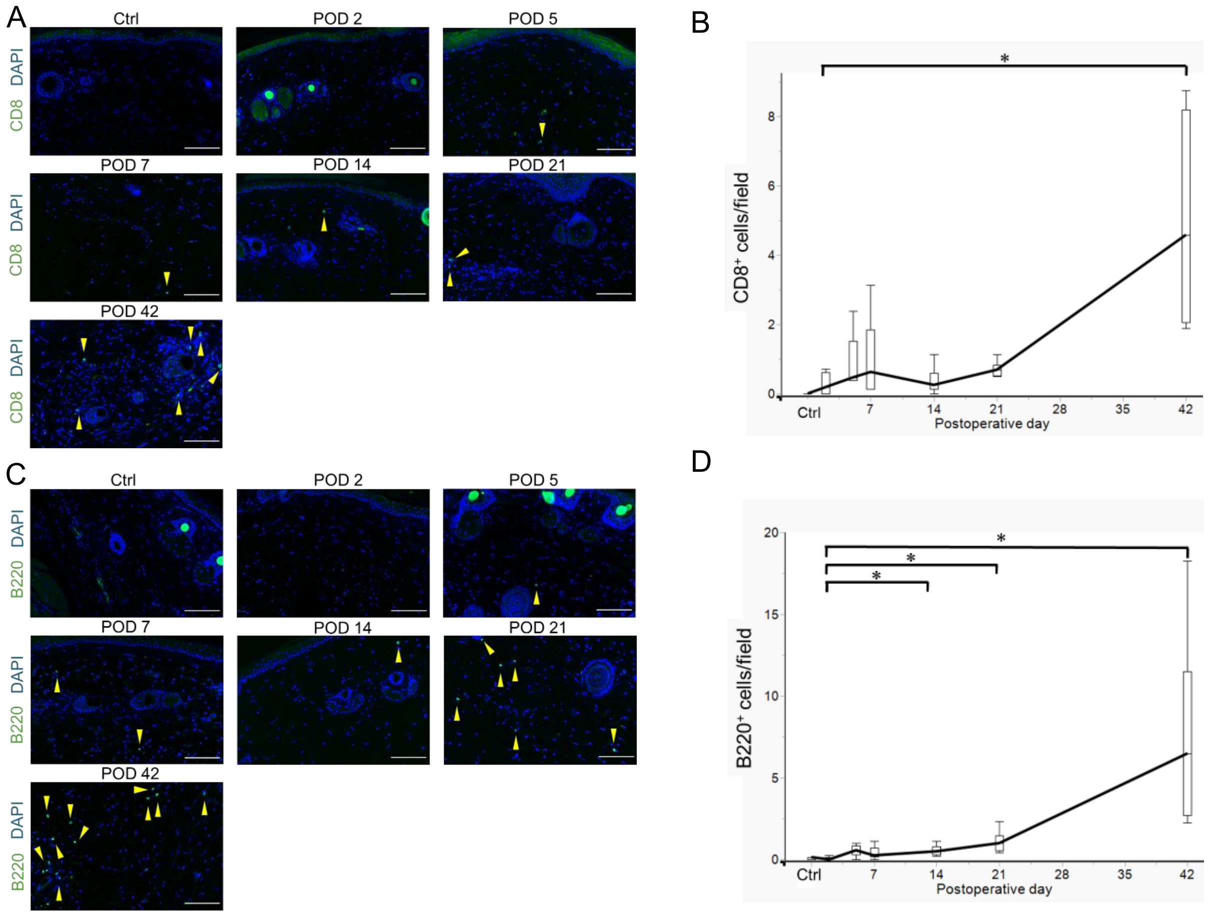

Figure S5. Infiltration of CD8^{+} T and B220^{+} B cells in the tail lymphedema tissue in non-operated controls (Ctrl) and on postoperative day (POD) 2, 5, 7, 14, 21, and 42.

A. Representative images of CD8^{+} T cells (green) infiltrating in the lymphedema tissues (Ctrl and POD 2, 5, 7, 14, 21, and 42). Yellow arrows indicate CD8^{+} T cells.

B. Variation in the numbers of CD8^{+} T cells per field (8 fields/mouse) infiltrating the lymphedema tissue (Ctrl: n = 3; POD 2, 5, 7, 14, and 21: n = 8; POD 42: n = 6). Boxes represent 50 % of the data, with medians (lines), interquartile ranges (whiskers). The Steel–Dwass test was used to determine the time points that were significantly different from POD 2. *$P < 0.05$.

C. Representative images of B220^{+} B cells (green) infiltrating in the lymphedema tissues (Ctrl and POD 2, 5, 7, 14,

21, and 42). Yellow arrows indicate $B220^+$ B cells.

D. Variation in the numbers of $B220^+$ B cells per field (8 fields/mouse) infiltrating in the lymphedema tissue (Ctrl: n = 3; POD 2, 5, 7, 14, and 21: n = 8; POD 42: n = 6). Boxes represent 50 % of the data, with medians (lines), interquartile ranges (whiskers). The Steel–Dwass test was used to determine the time points that were significantly different from POD 2. *$P < 0.05$.

Nuclei in (A) and (C) were stained with 4′,6-diamidino-2-phenylindole (DAPI) (blue). Scale bar = 100 μm

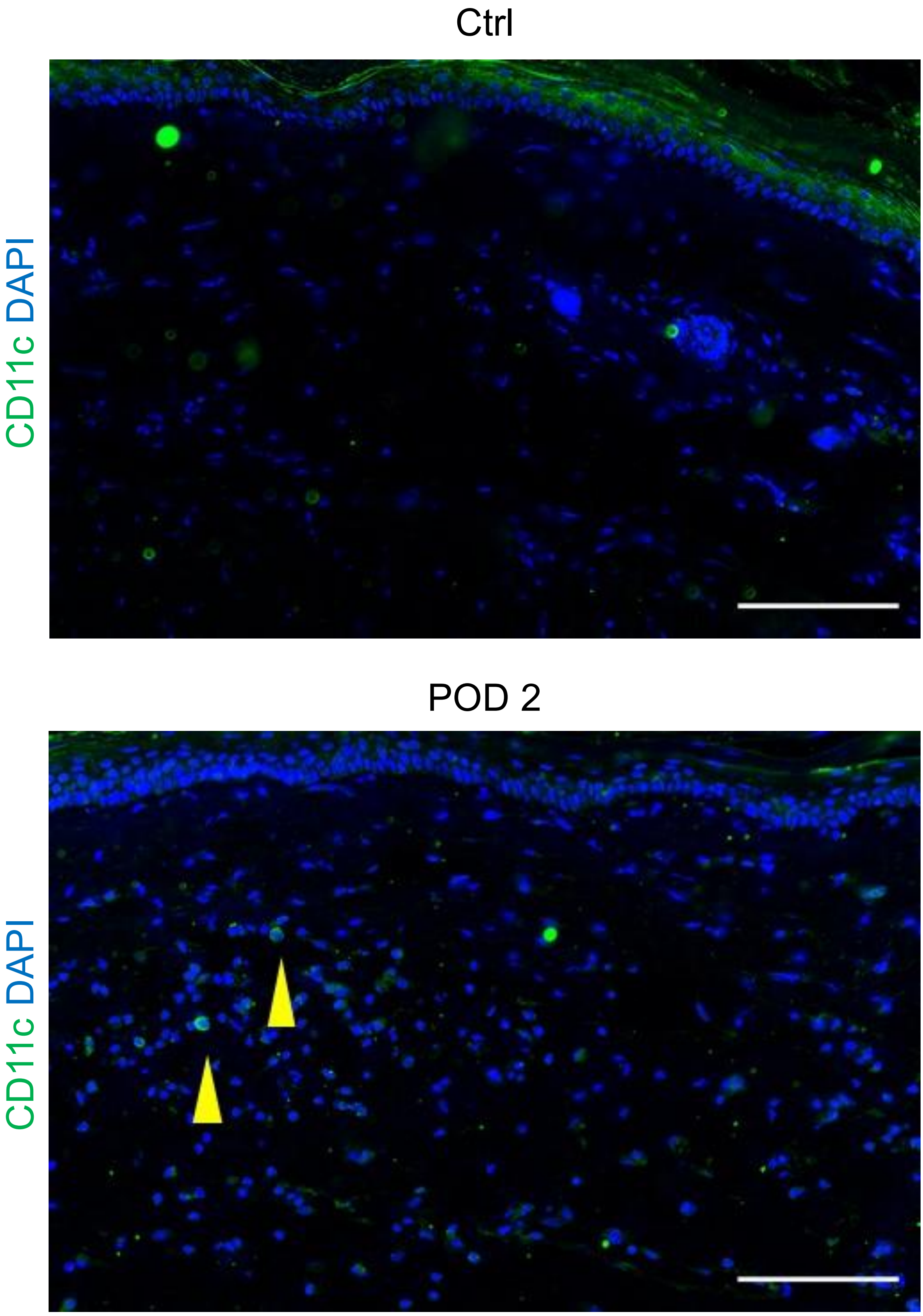
Ctrl
CD11c DAPI
POD 2
CD11c DAPI

POD 5

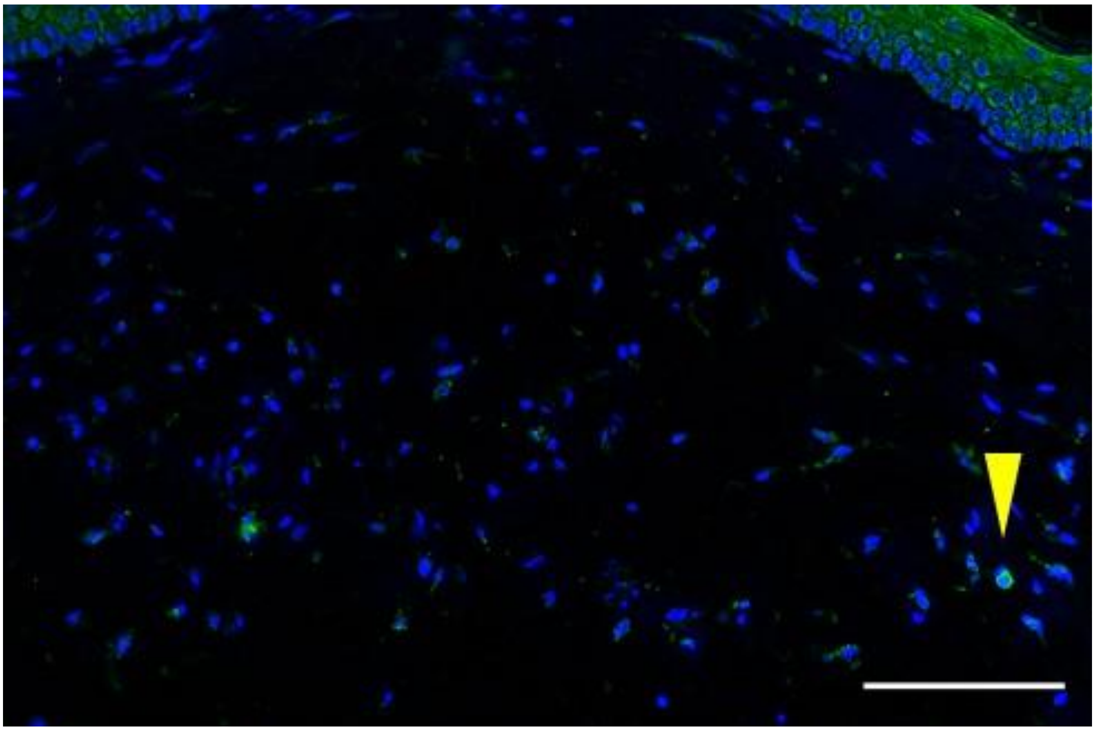

POD 7

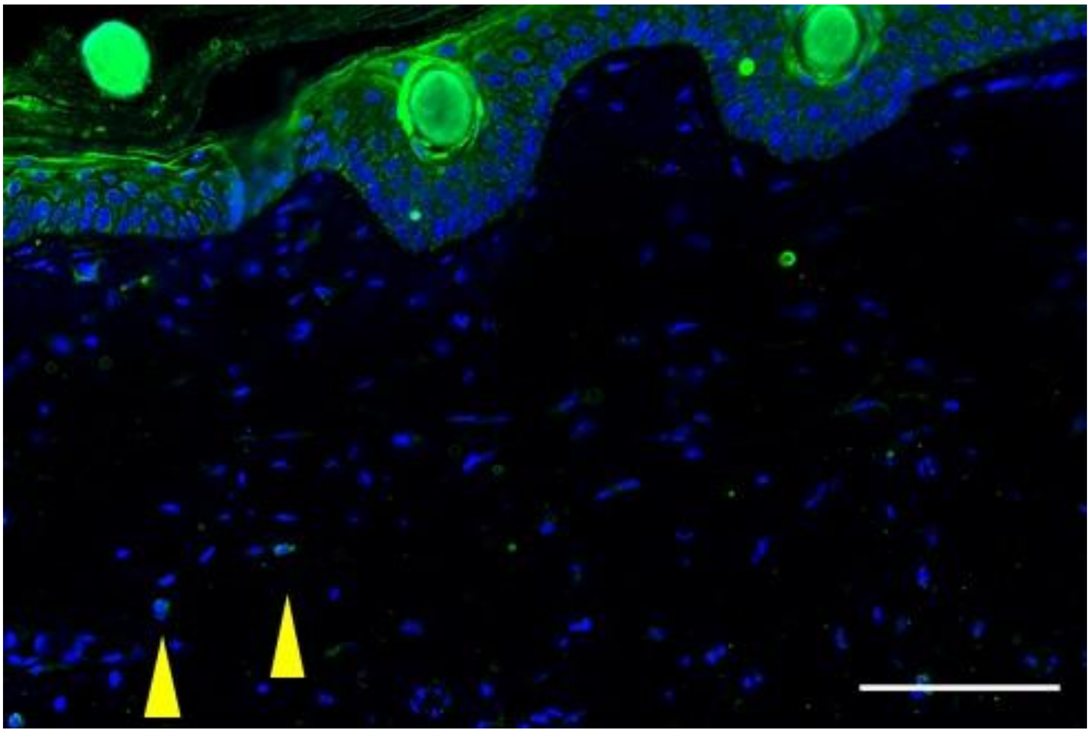

POD 14

CD11c DAPI

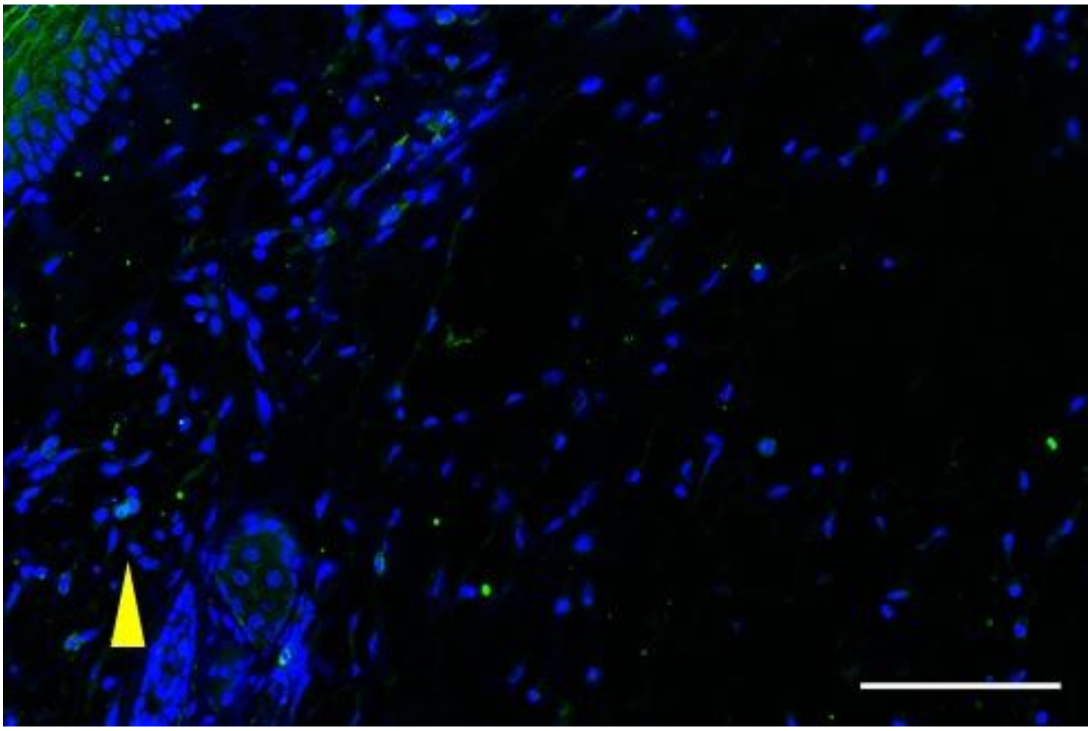

POD 21

CD11c DAPI

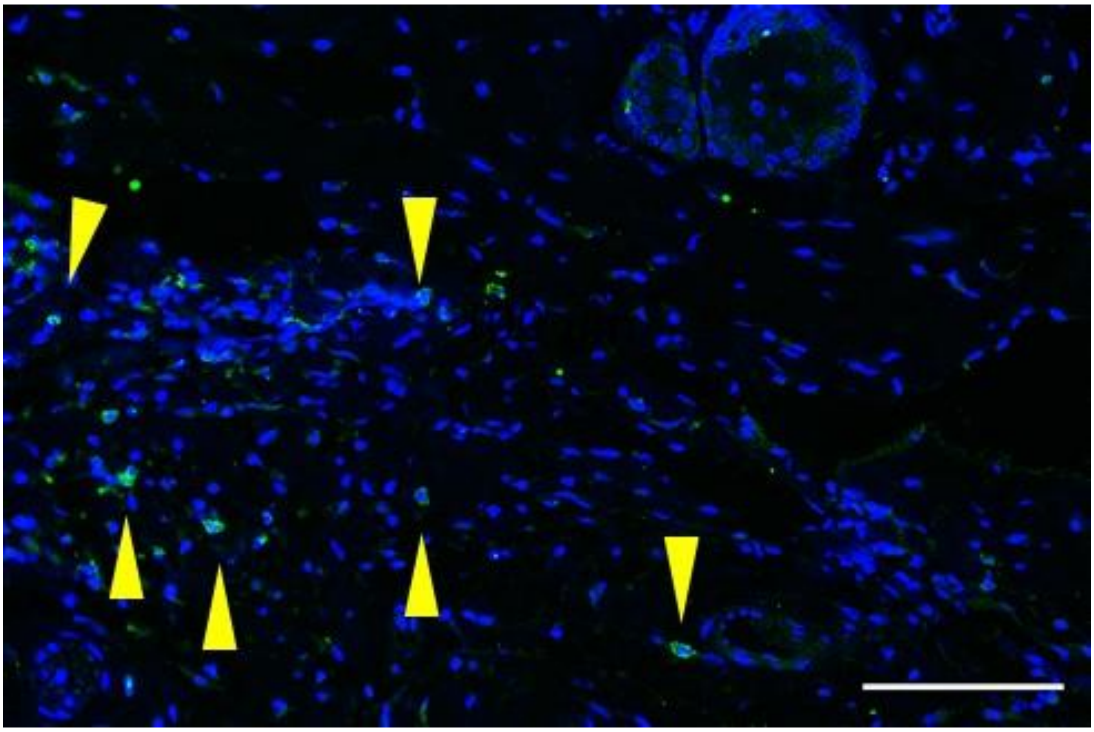

POD 42

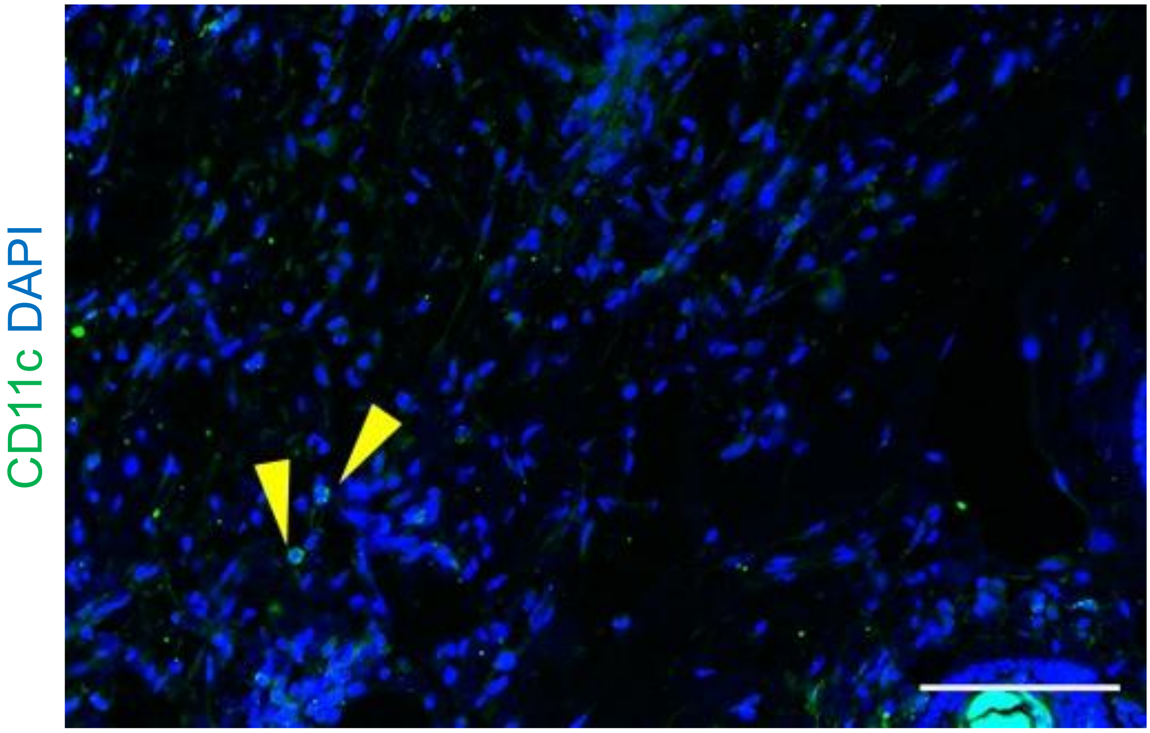

Figure S6. Enlarged views of Fig. 3A.

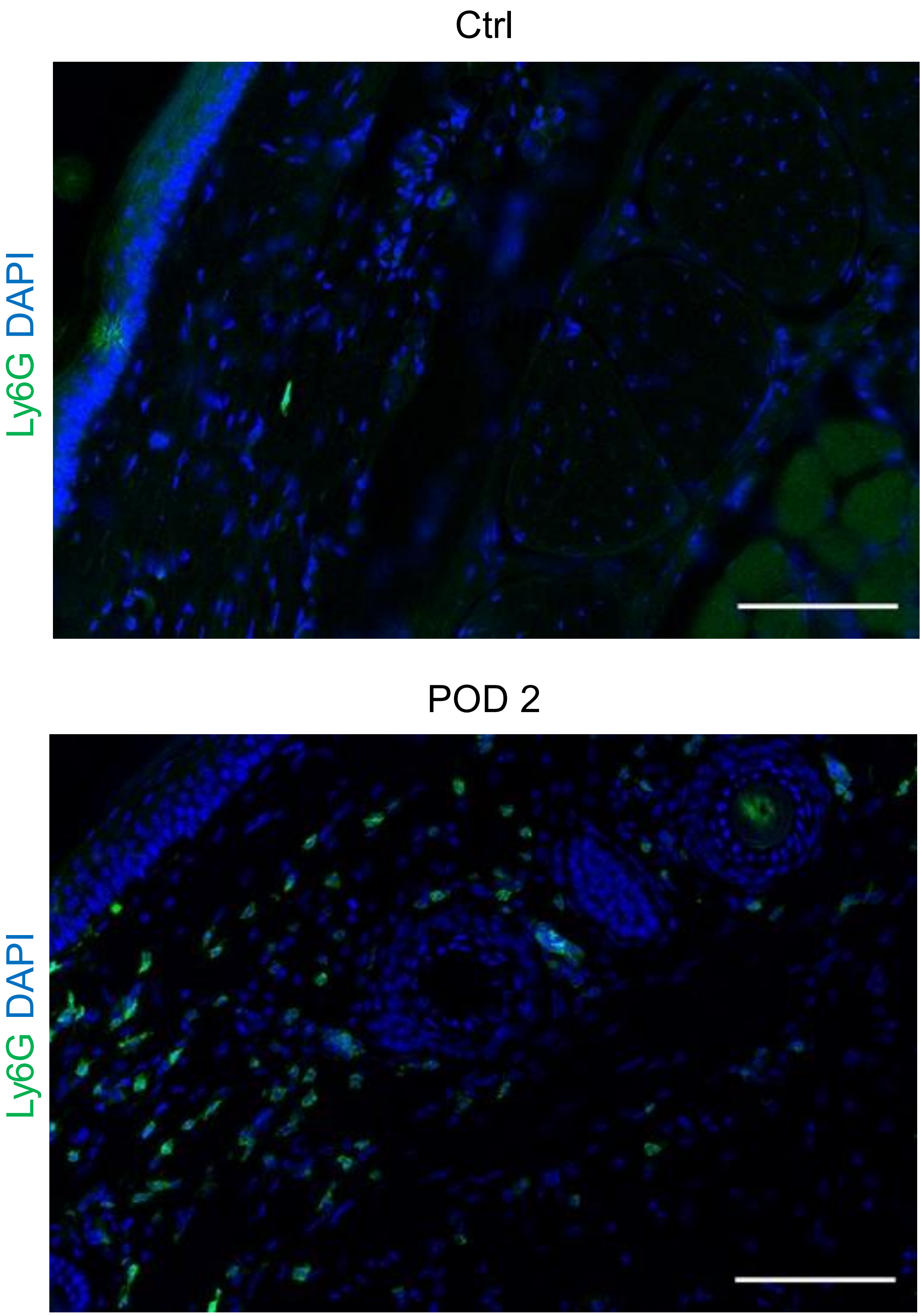
Ctrl
Ly6G DAPI
POD 2
Ly6G DAPI

POD 5

Ly6G DAPI

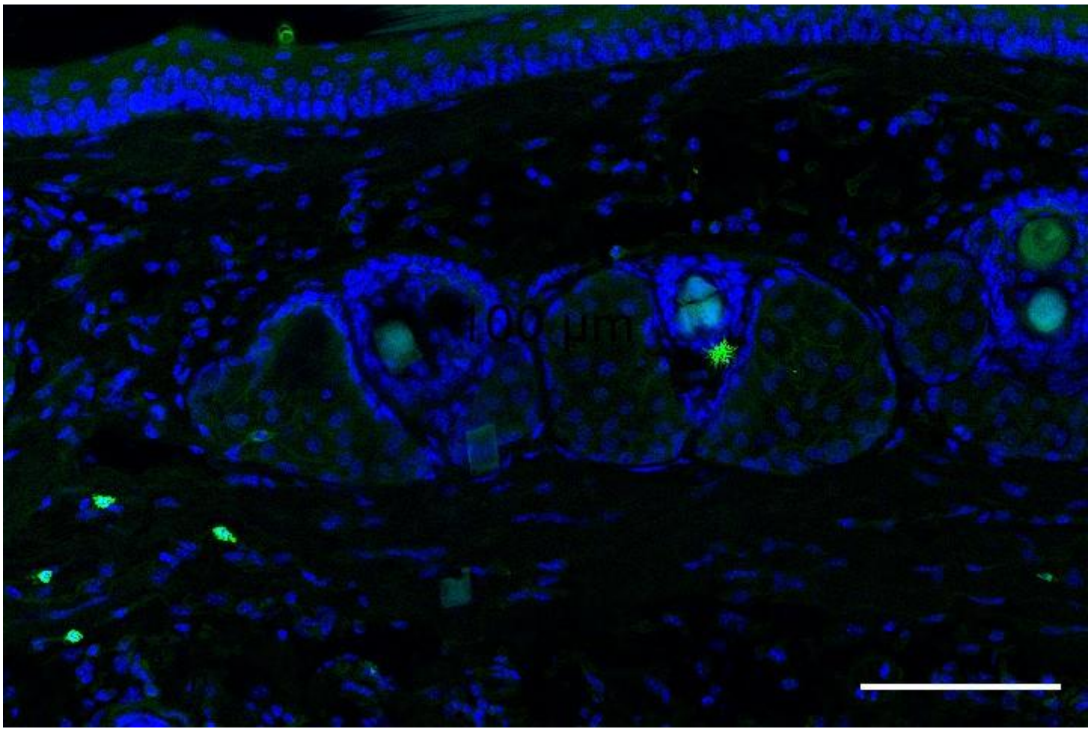

POD 7

Ly6G DAPI

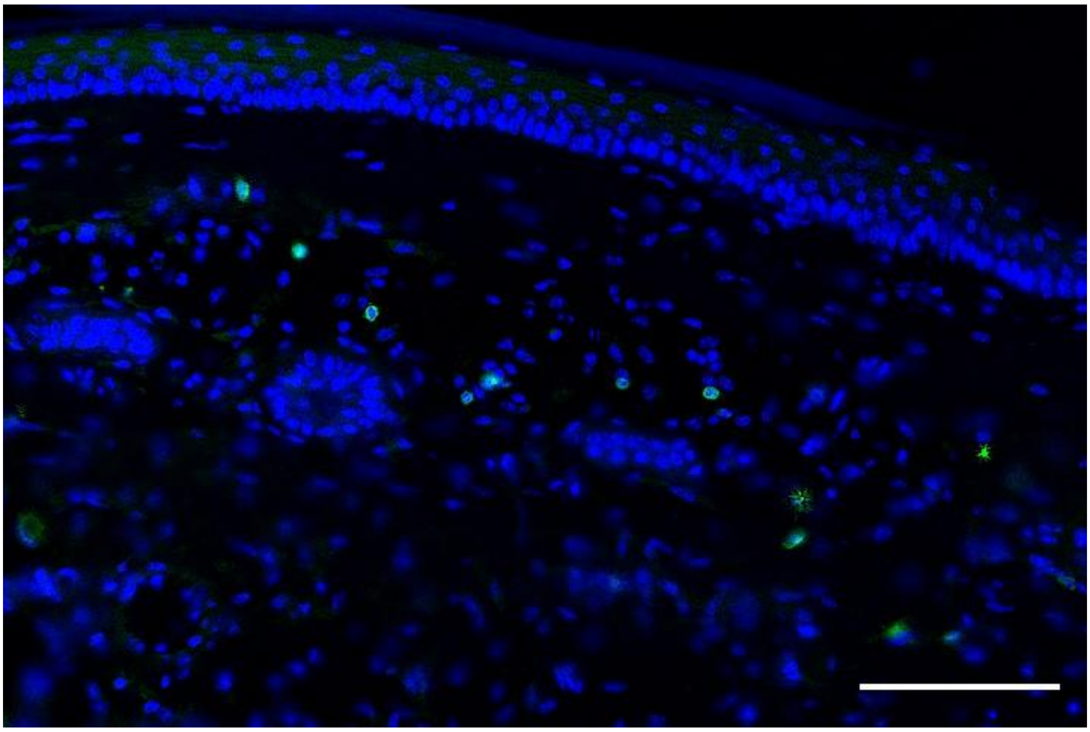

POD 14

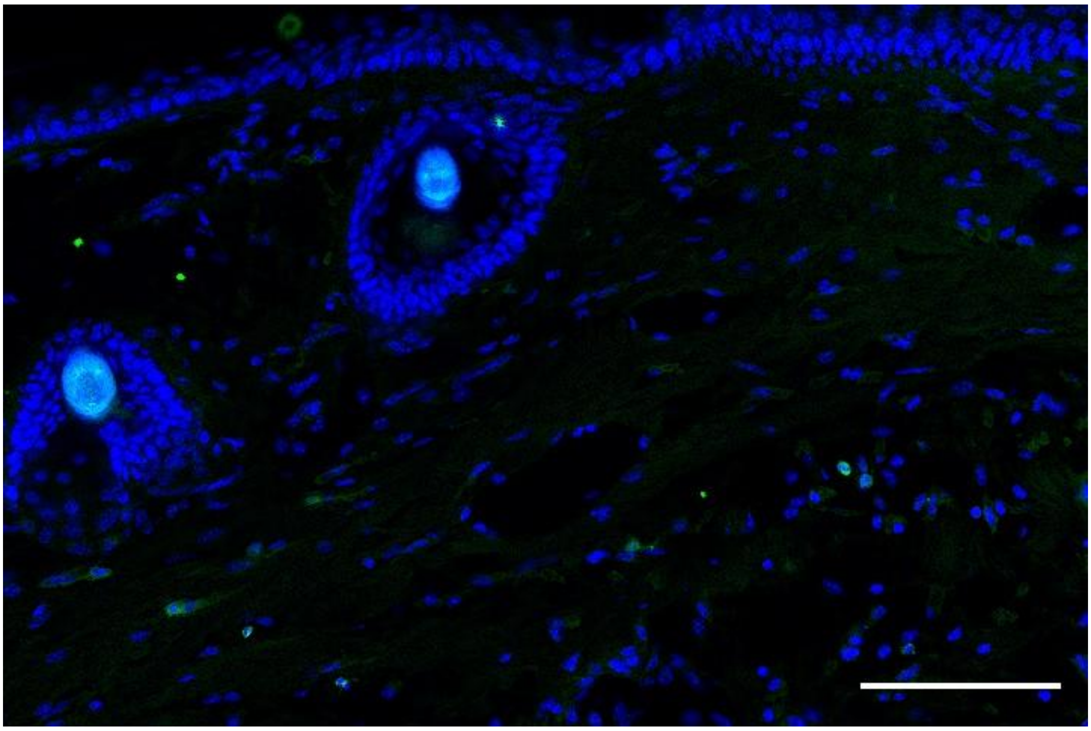

POD 21

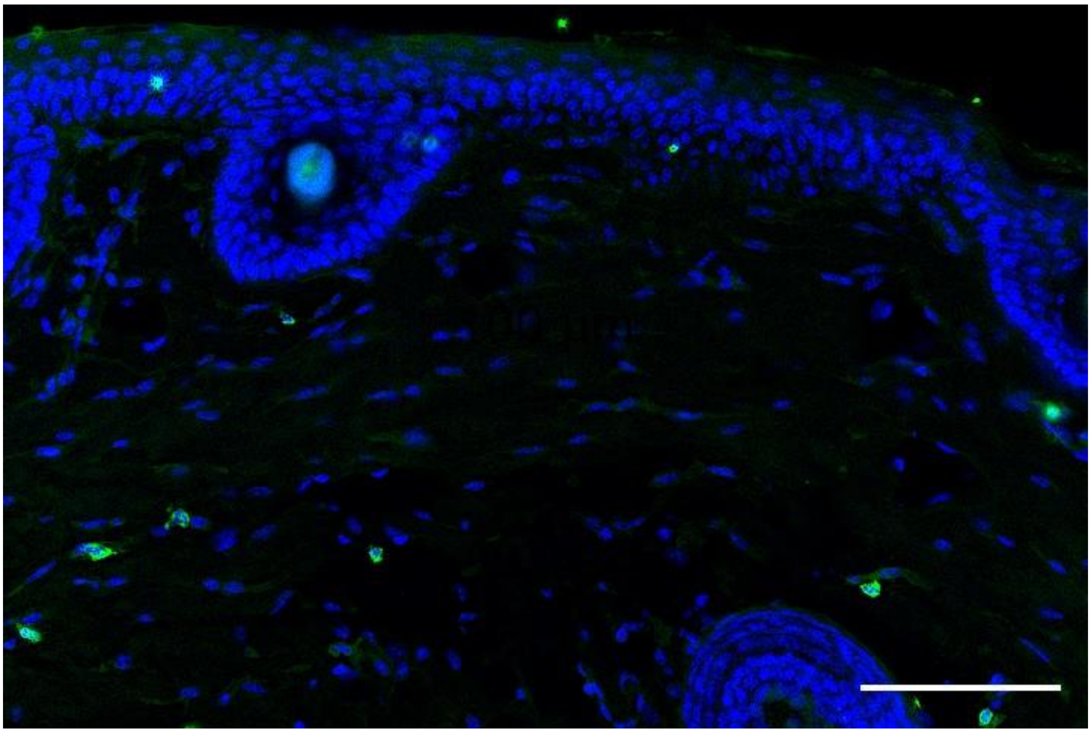

POD 42

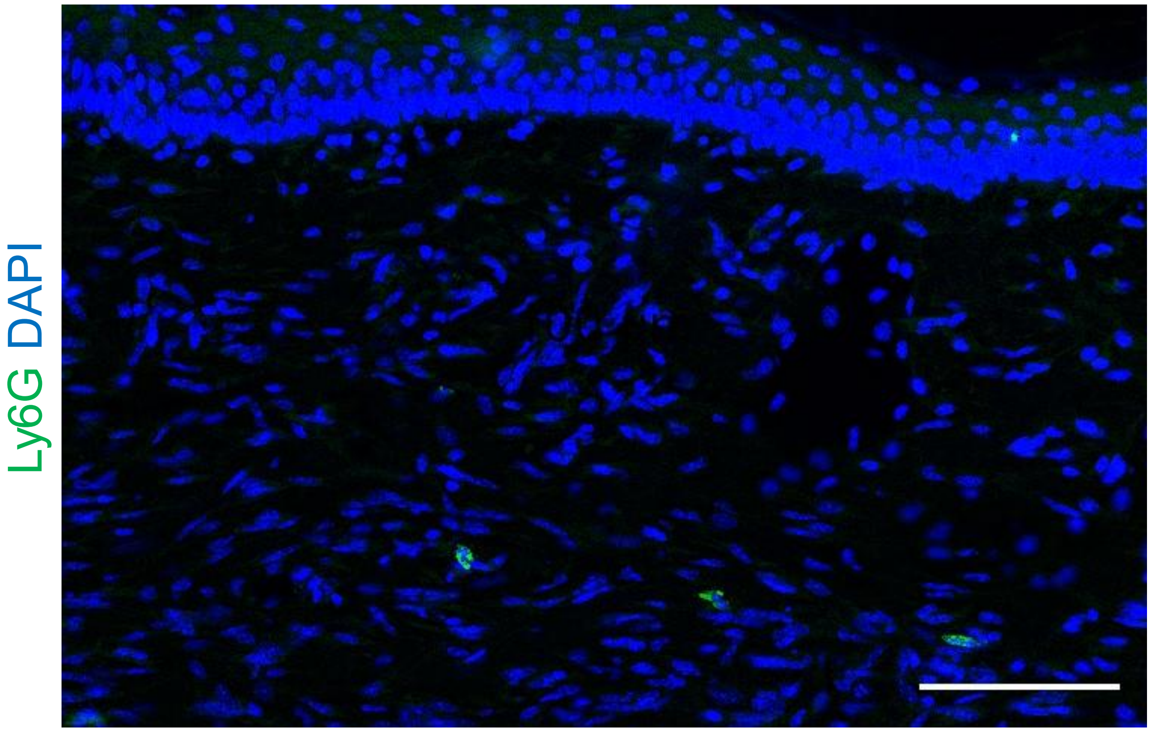

Figure S7. Enlarged views of Fig. 5A.

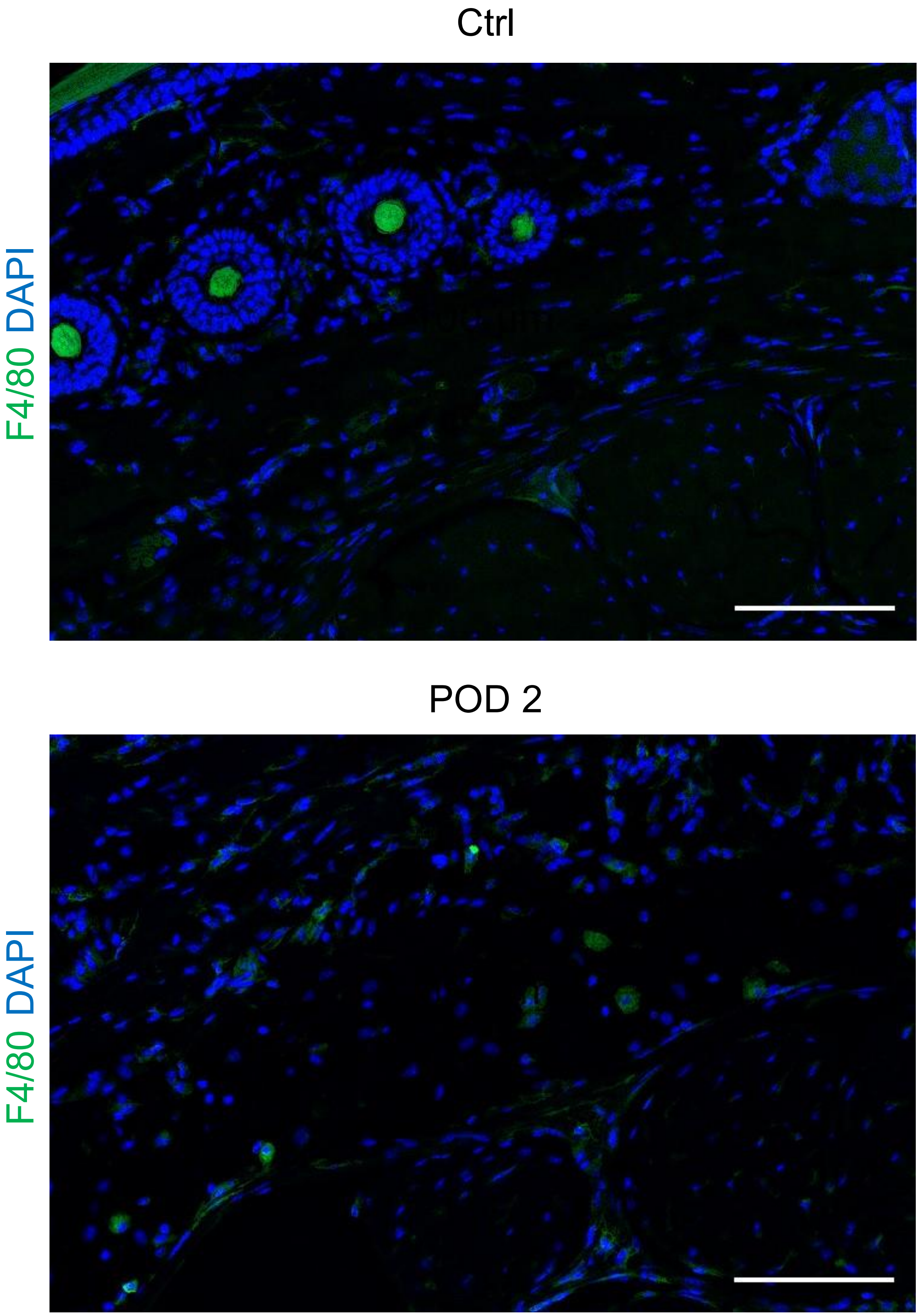
Ctrl
F4/80 DAPI
POD 2
F4/80 DAPI

POD 5

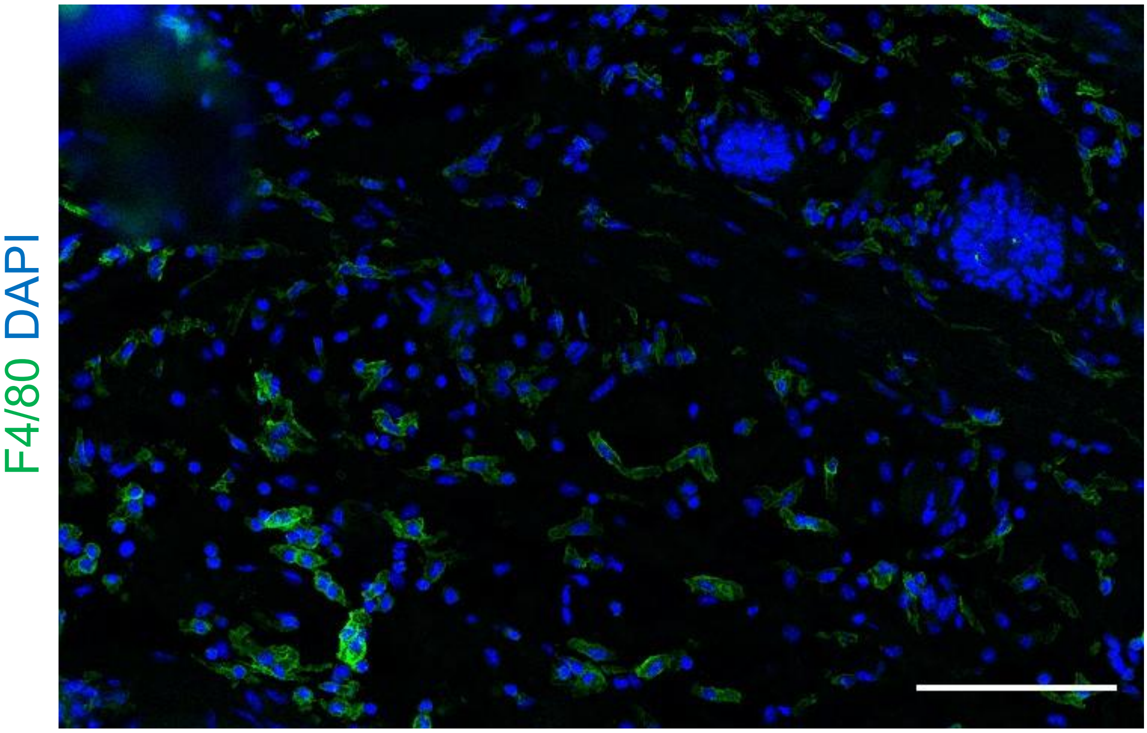

POD 7

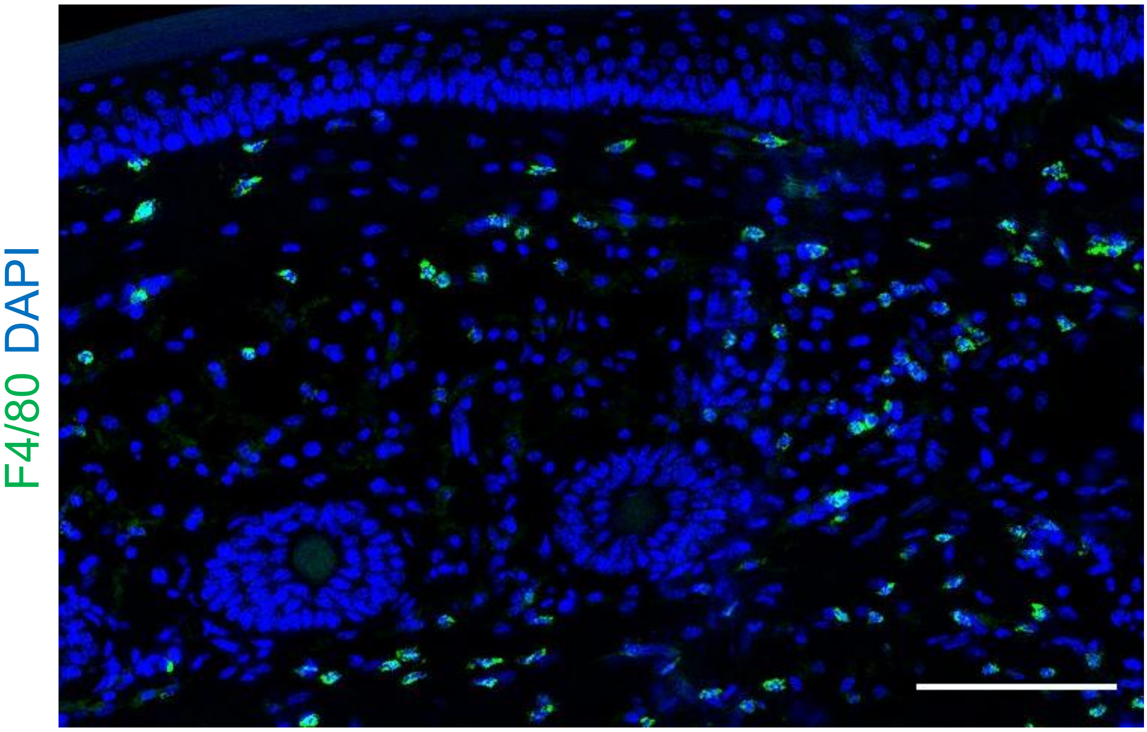

POD 14

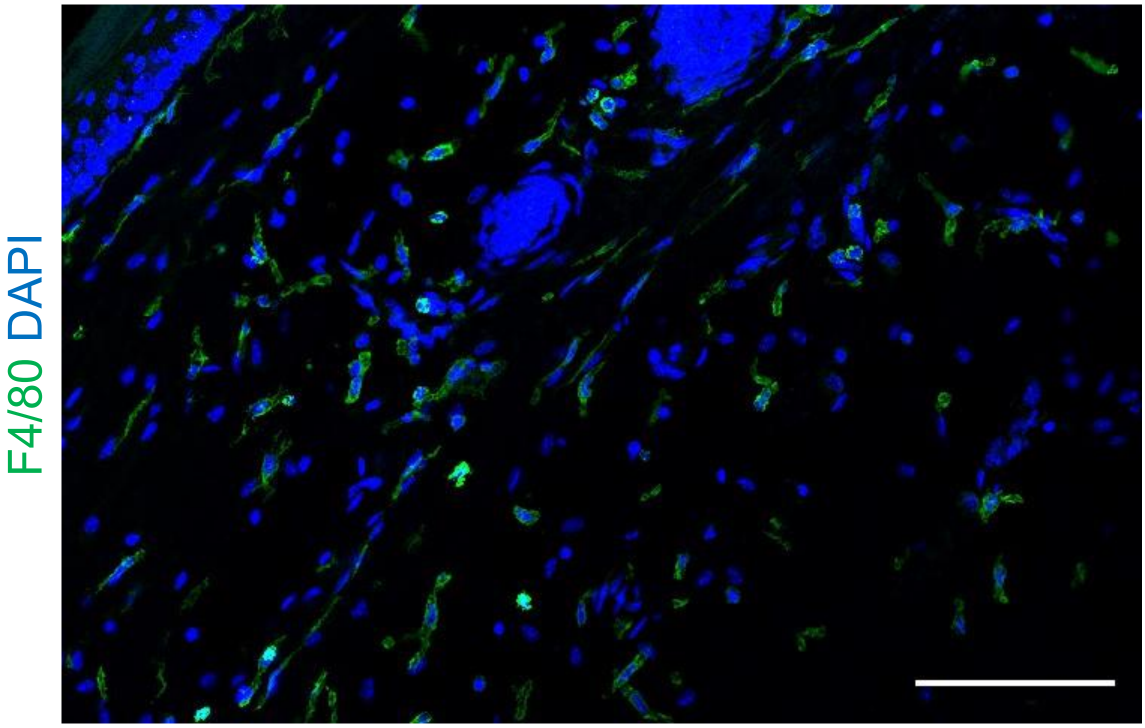

POD 21

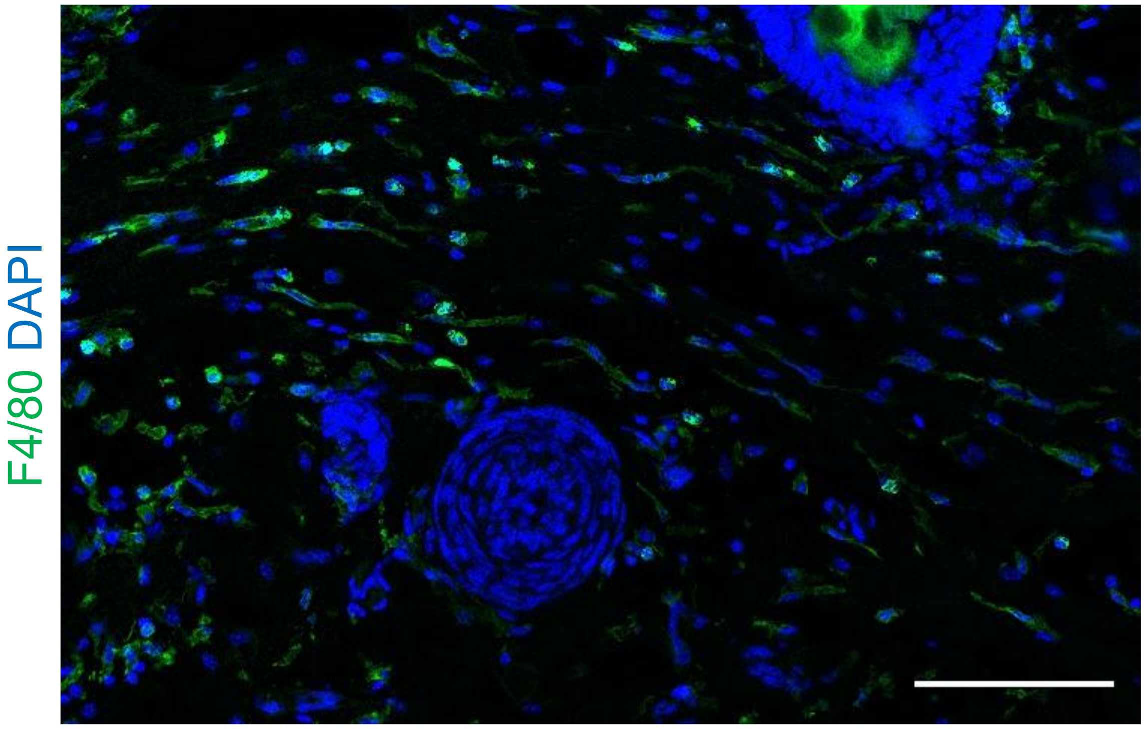

POD 42

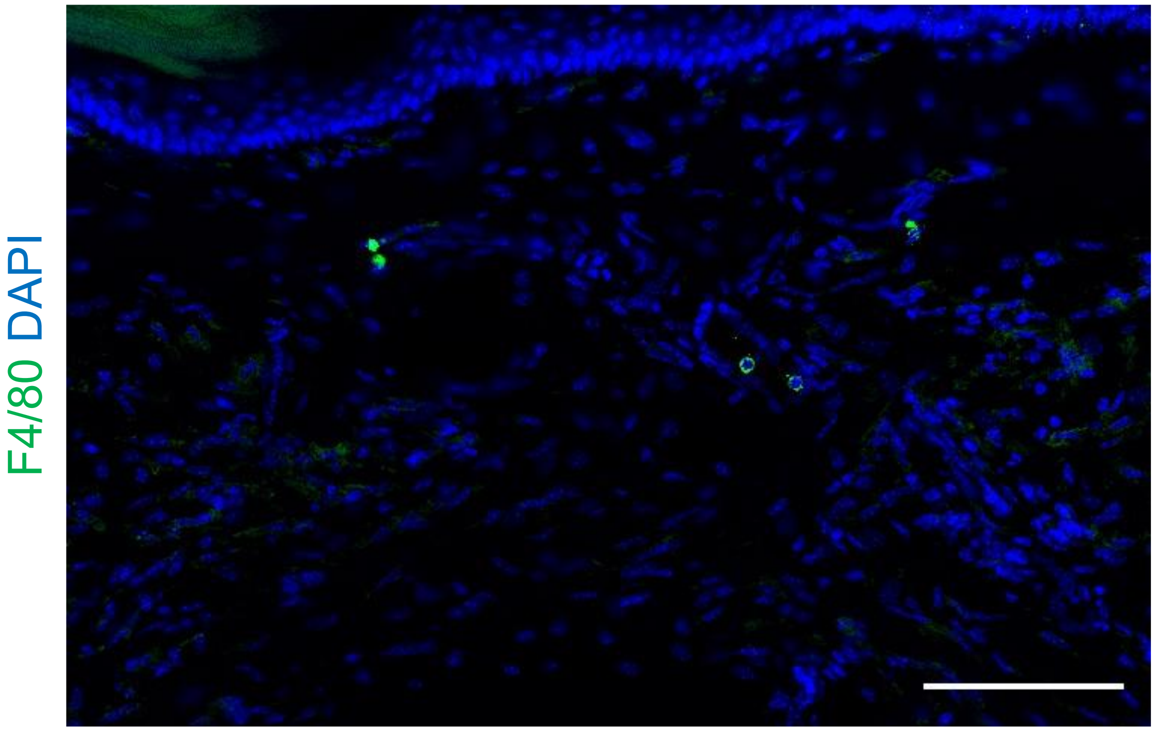

Figure S8. Enlarged views of Fig. 5C.

A

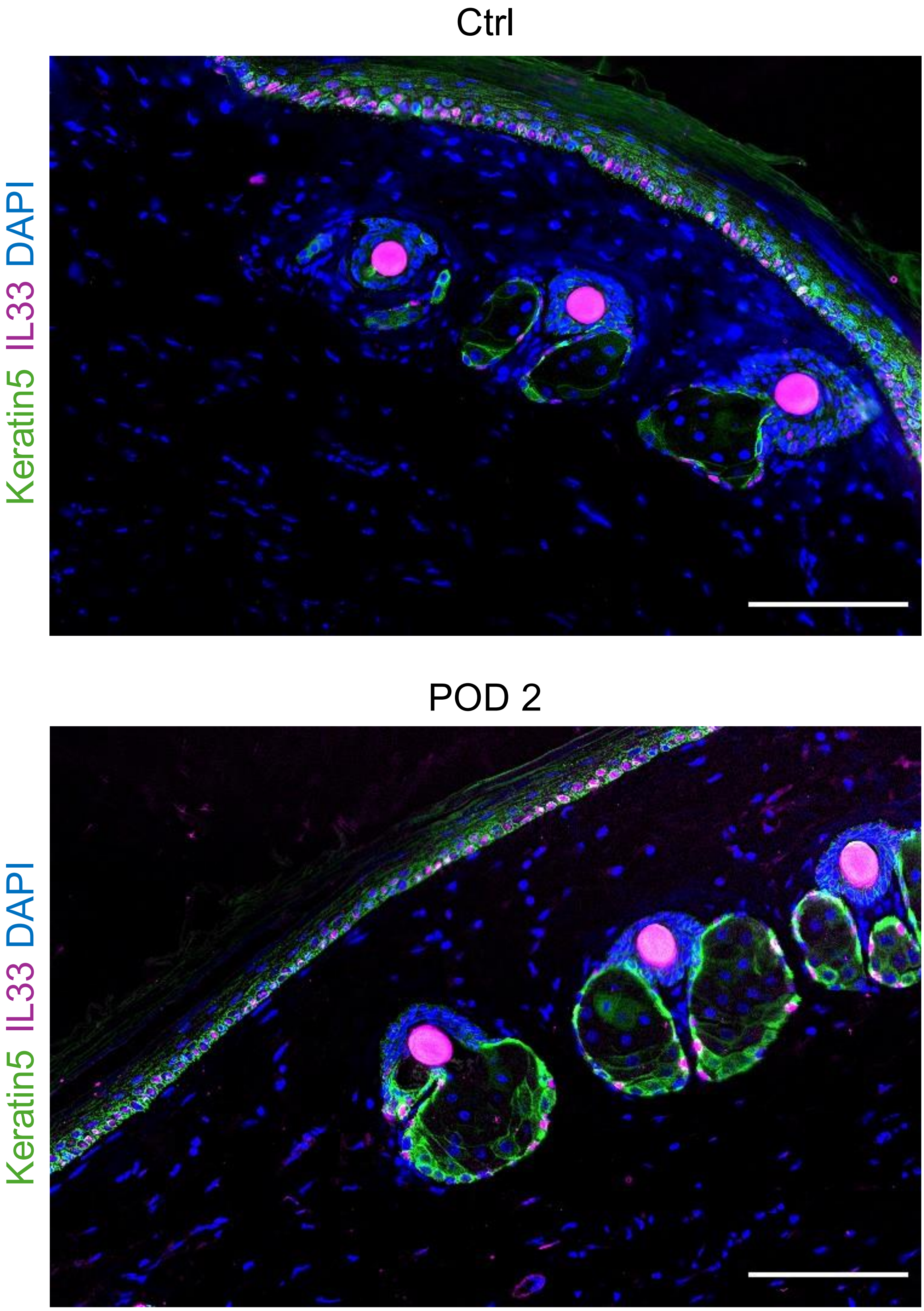

POD 5

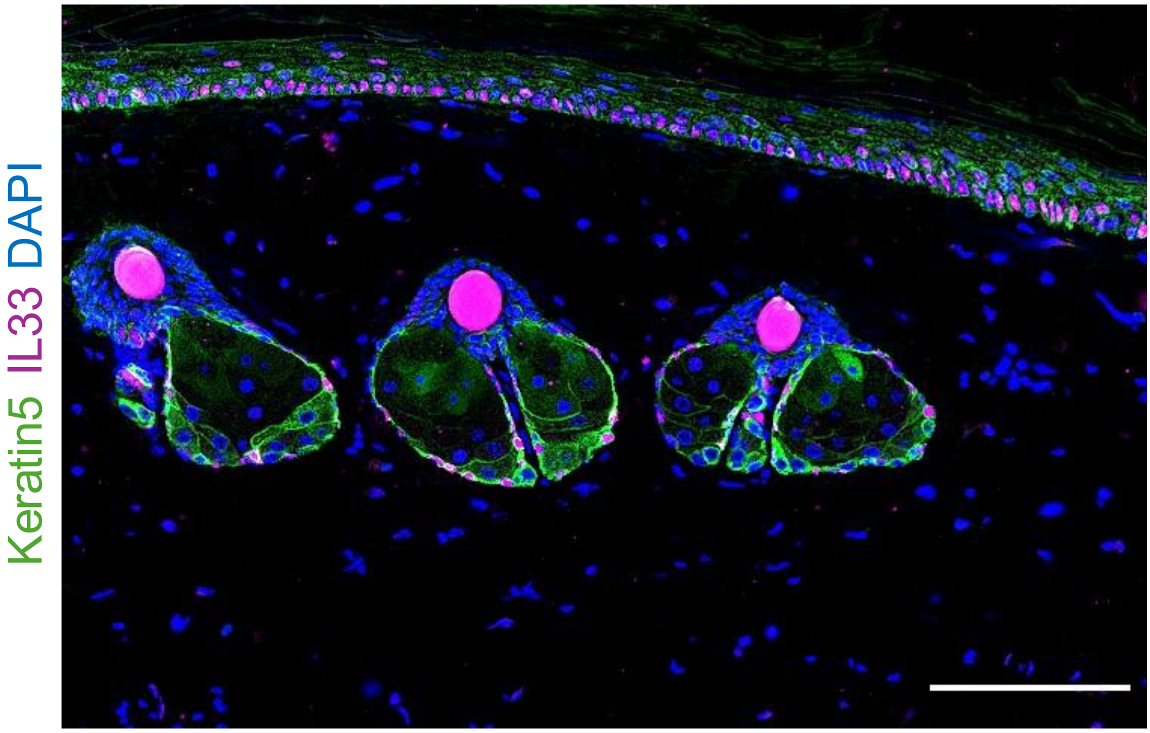

POD 7

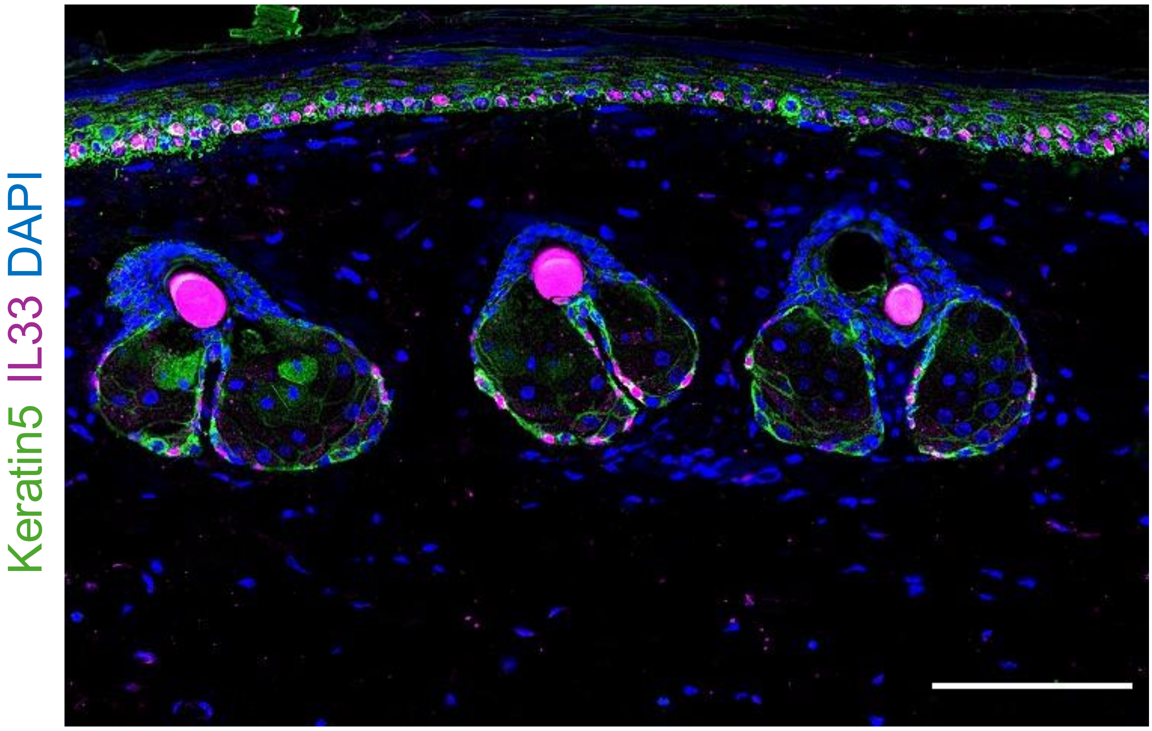

POD 14

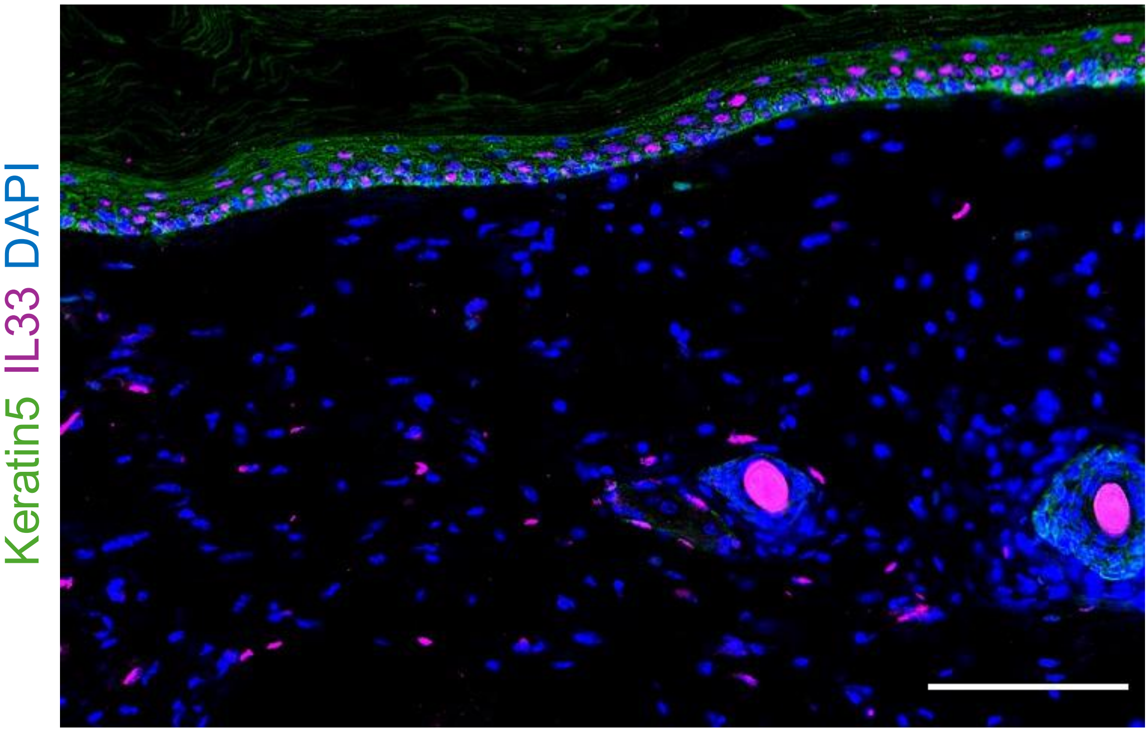

POD 21

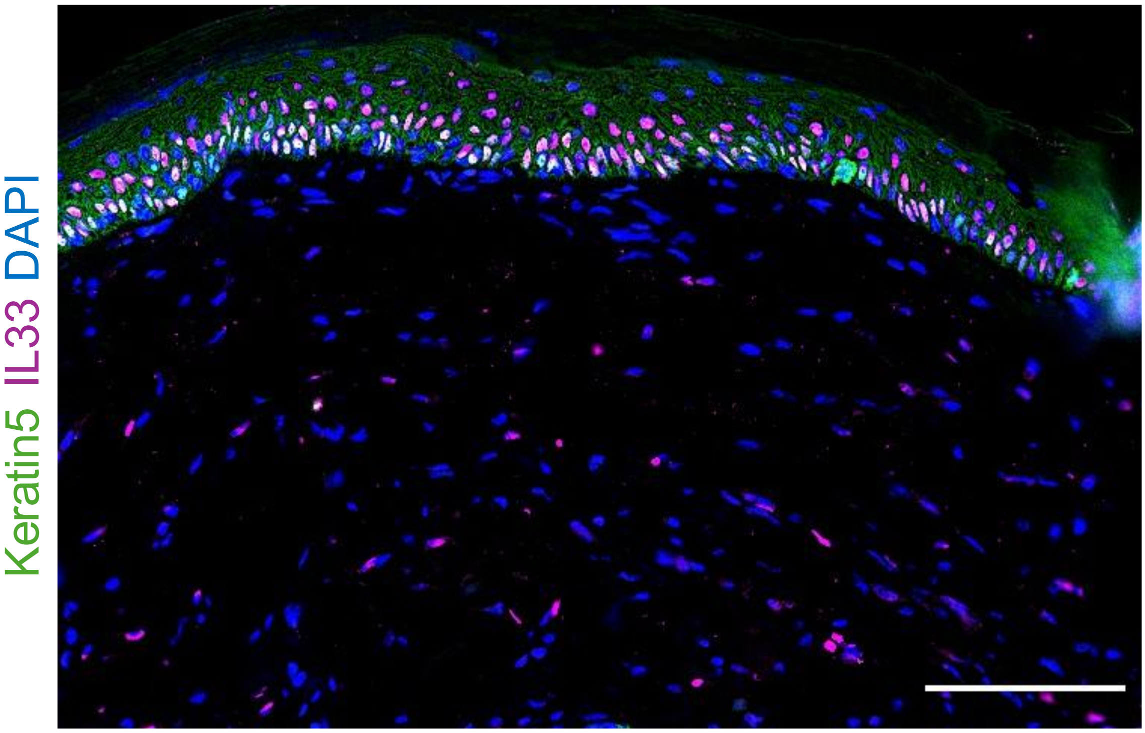

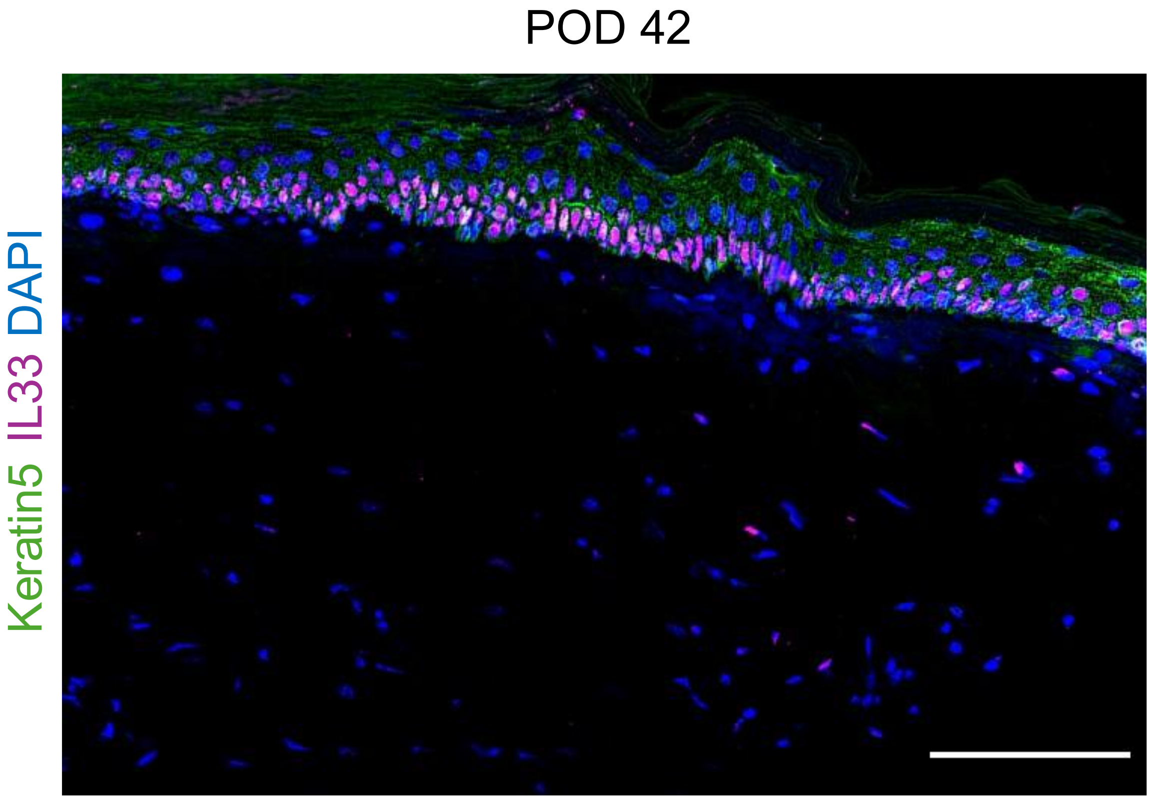

B

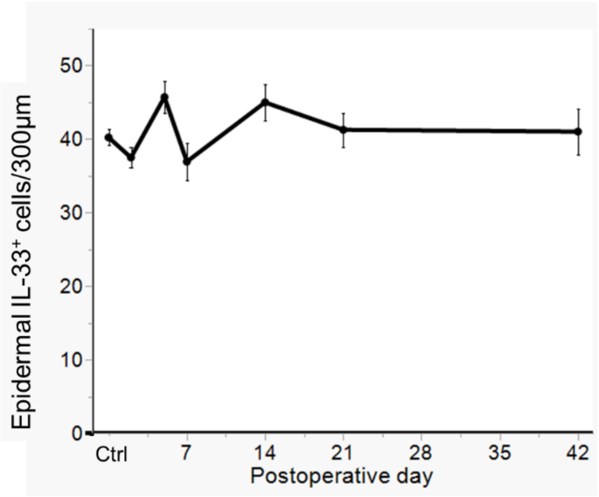

Figure S9. Epidermal interleukin (IL)-33^{+} cells in the tail lymphedema tissue.

A. Representative images of IL-33 (magenta) in epidermal basal lamina stained by keratin 5 (green) in the lymphedema tissues (Ctrl and POD 2, 5, 7, 14, 21, and 42).

Nuclei were stained with 4′,6-diamidino-2-phenylindole (DAPI) (blue). Scale bar = 100 μm

B. Variation in the numbers of epidermal IL-33^+ cells per 300 μm (8 fields/mouse) in the lymphedema tissue (Ctrl: n = 3; POD 2, 5, 7, 14, and 21: n = 8; POD 42: n = 6). Data are shown as the means ± standard error. The Tukey test revealed no significant differences between the time points.

Vimentin IL-33 DAPI

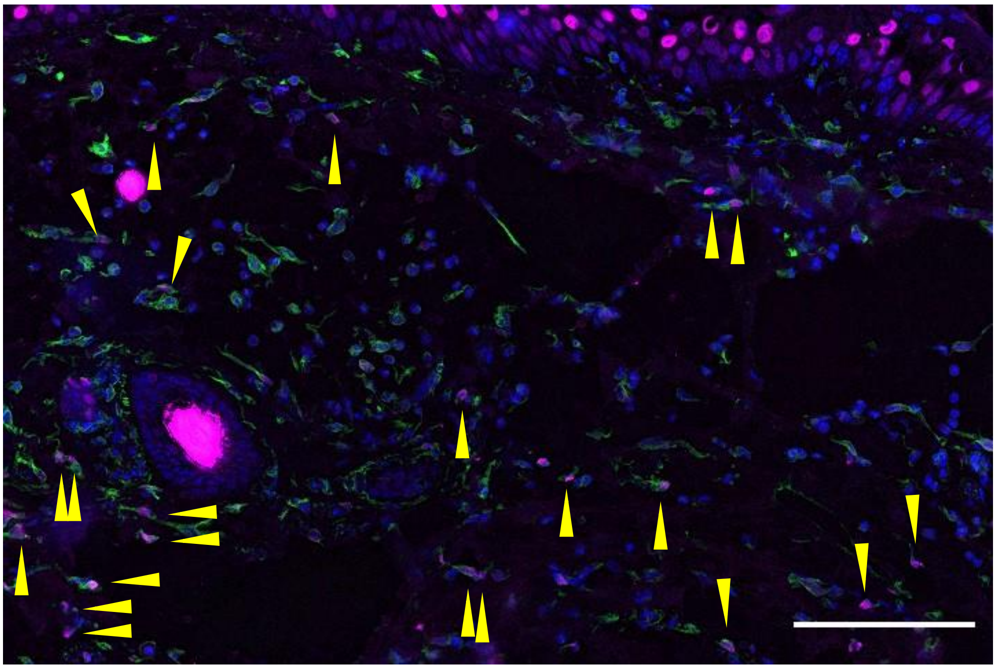

HSP47 IL-33 DAPI

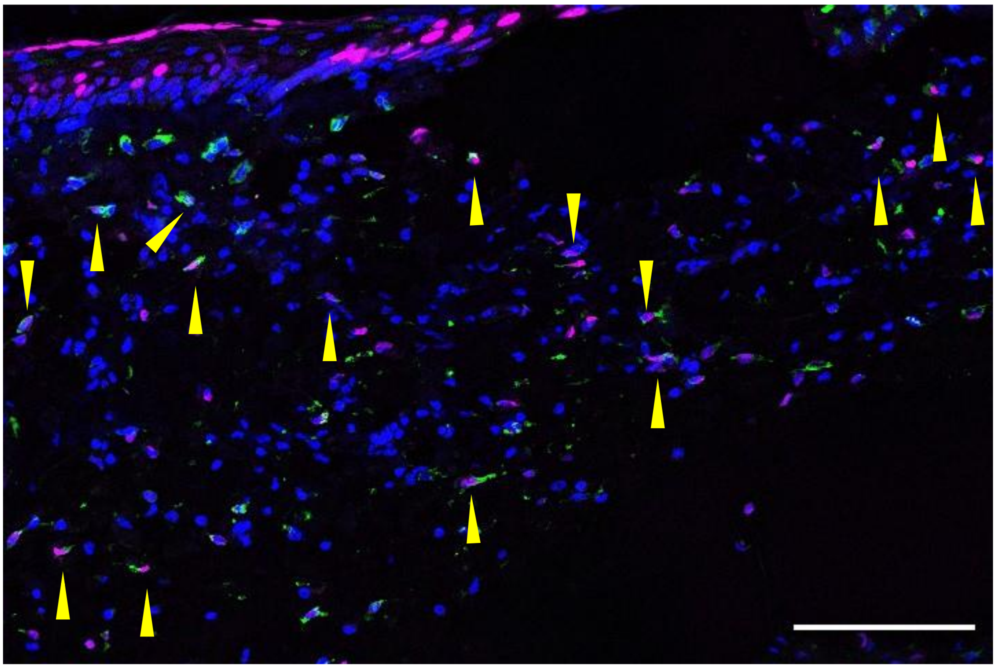

S100A4 IL-33 DAPI

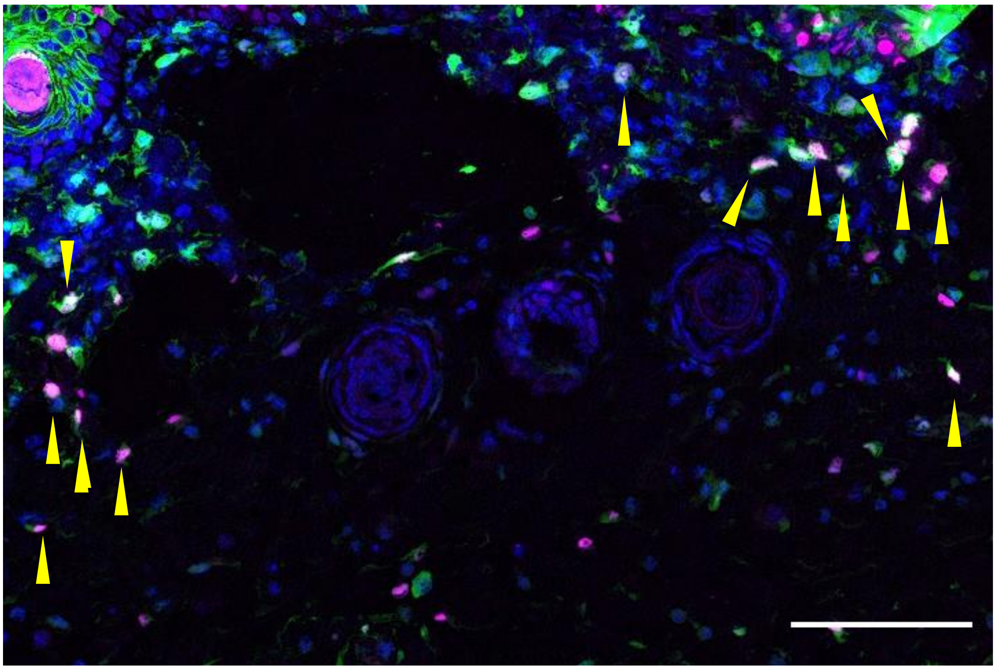

Figure S10. Enlarged views of Fig. 7I, L and O.

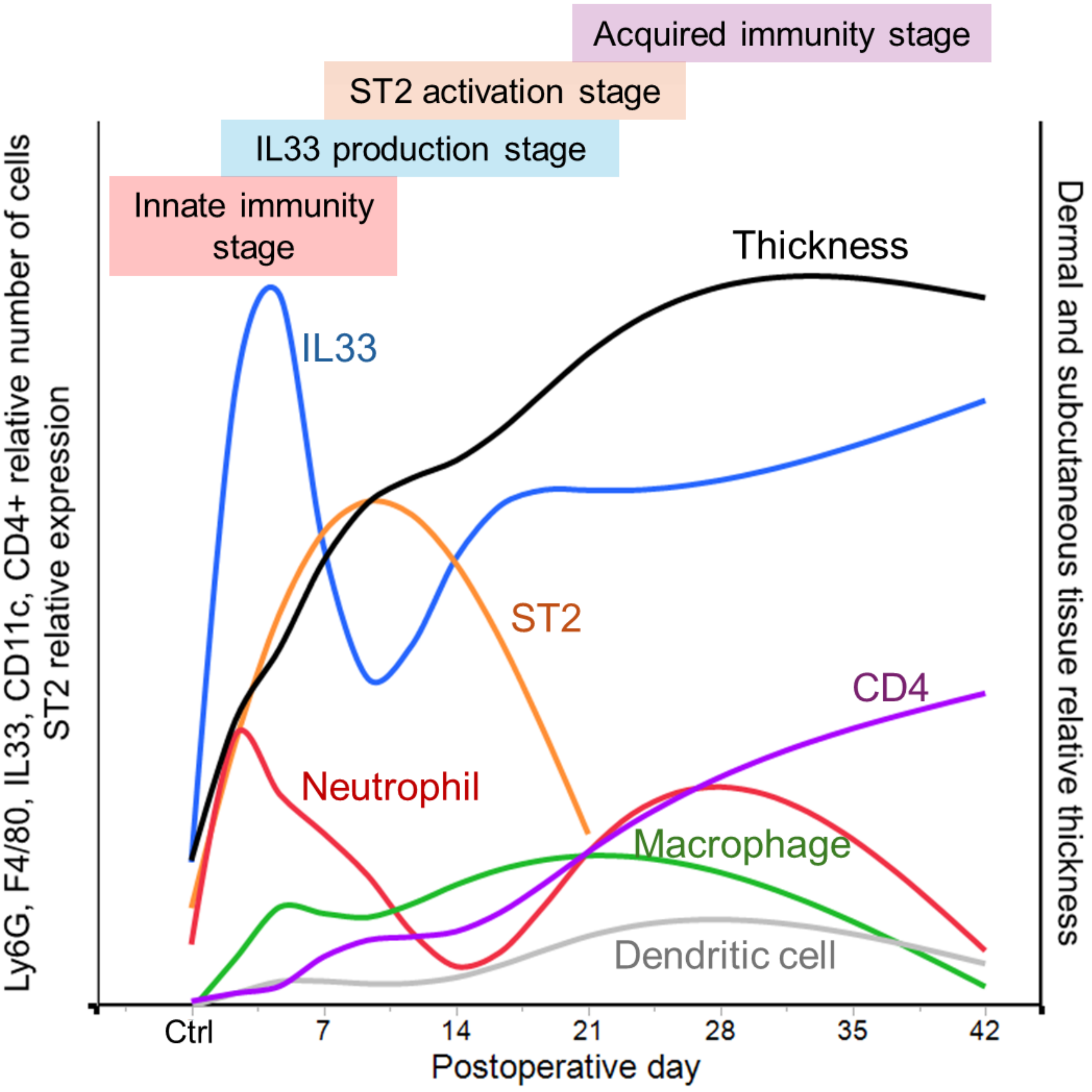

Figure S11. Dynamics of immune cells in lymphedema development

Postoperative lymphedema progresses via innate immunity, interleukin (IL)-33 production, suppression of tumorigenicity 2 (ST2) activation, and acquired immunity.

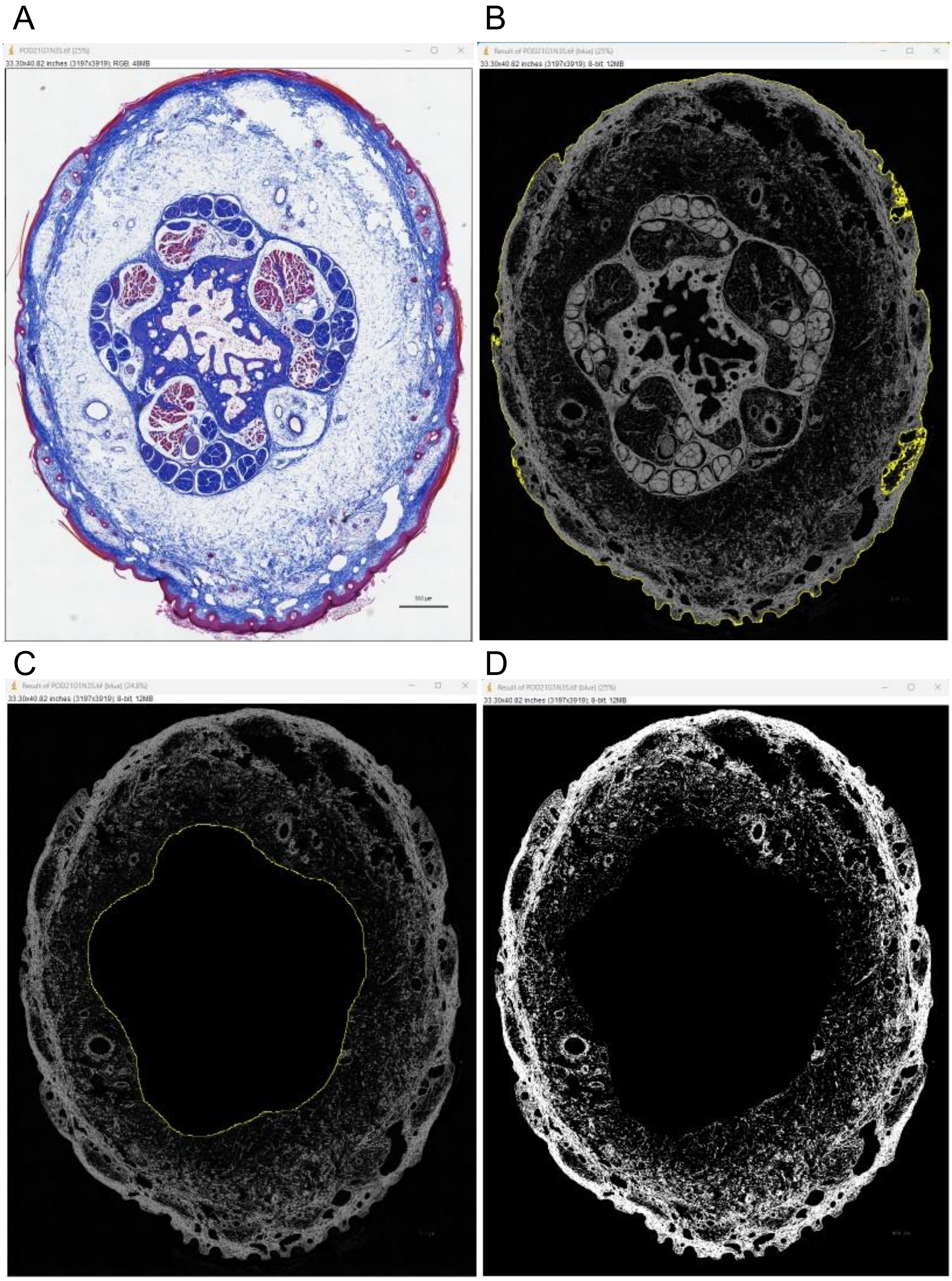

Figure S12. Procedure for measurement of fibrotic tissue area and percentage in the dermal and subcutaneous regions.

A. Representative images of POD 21 lymphedema tissues stained with Masson's trichrome.

B. The images were split into RGB channels and the red channel was subtracted from the blue channel. The outer border is outlined in yellow.

C. Image that removed bone and muscle regions (yellow).

D. The lower threshold was set at 50%, and the area and percentage of fibrotic tissue in the dermal and

subcutaneous tissues were measured.

Table S1. The average numbers of immune cell per field each mouse

Postoperative day	Mouse No.	CD4+ cells/field	CD11c+ cells/field	LY6G+ cells/field	F480+ cells/field	Dermal and subcutaneous IL33+ cells/field	Epidermal IL33+ cells/300μm	CD8+ cells/field	B220+ cells/field	CD4/Foxp3+ cells/field	%Treg
Control	1	0.1	0.0	0.0	0.0	8.4	39.0	0.0	0.0	0.0	0.0
Control	2	0.1	0.0	0.5	0.0	3.6	39.3	0.0	0.1	0.0	0.0
Control	3	0.0	0.0	0.0	0.0	2.6	42.4	0.0	0.1	0.0	0.0
2	1	0.4	0.1	3.4	0.7	30.5	37.8	0.3	0.0	0.0	0.0
2	2	0.9	0.3	2.7	1.0	19.3	44.8	0.0	0.0	0.1	14.3
2	3	0.3	0.2	1.8	1.8	9.9	39.7	0.0	0.0	0.0	0.0
2	4	2.1	0.0	20.5	0.1	44.4	36.0	0.1	0.3	0.6	29.4
2	5	2.1	2.3	17.9	4.2	51.0	38.3	0.7	0.0	0.4	17.6
2	6	0.4	0.6	6.7	1.9	46.0	37.8	0.7	0.1	0.3	66.7
2	7	1.1	0.5	57.4	0.6	29.8	32.3	0.0	0.0	0.4	33.3
2	8	2.4	0.4	26.5	4.8	35.9	33.4	0.4	0.1	0.3	10.5
5	1	1.4	2.0	2.5	0.5	47.0	41.0	0.4	0.6	0.9	63.6
5	2	0.5	1.7	1.0	4.3	13.0	42.3	0.4	0.0	0.5	100.0
5	3	3.5	1.1	12.4	16.7	47.0	52.0	0.4	0.3	1.8	50.0
5	4	1.5	2.5	1.4	10.2	43.3	45.2	0.5	0.5	0.5	33.3
5	5	0.9	1.0	5.8	5.1	37.5	50.8	0.4	0.4	0.3	28.6
5	6	1.0	1.4	26.5	6.8	41.4	35.3	2.4	0.8	0.4	37.5
5	7	2.1	1.0	16.9	5.5	58.6	54.0	1.8	0.9	1.0	47.1
5	8	2.9	1.6	10.1	1.8	40.3	45.1	0.8	1.0	1.3	43.5
7	1	2.6	1.5	2.1	9.0	8.5	30.9	0.1	0.3	1.3	47.6
7	2	1.9	1.9	5.0	5.4	12.1	36.0	0.3	0.3	0.5	26.7
7	3	4.4	0.4	2.8	1.3	26.9	29.0	0.1	0.3	1.0	22.9
7	4	2.3	0.9	17.5	3.3	7.1	30.6	0.1	0.0	1.4	61.1
7	5	10.6	3.1	15.8	1.5	40.6	40.8	3.1	1.1	6.9	64.7
7	6	6.6	1.1	7.0	10.8	29.8	50.4	1.0	0.6	5.3	79.2
7	7	7.3	1.3	8.9	1.1	32.8	42.9	1.8	0.1	5.3	72.4
7	8	4.8	0.8	24.8	6.5	31.8	35.0	1.9	0.8	2.9	60.5
14	1	5.9	0.5	1.4	8.8	16.6	38.8	1.1	0.5	2.8	46.8
14	2	3.3	0.3	1.6	8.8	27.5	40.4	0.6	0.9	2.3	69.2
14	3	3.7	0.8	4.0	9.6	21.8	55.7	0.5	0.2	2.2	60.0
14	4	10.7	2.5	1.0	3.4	30.1	43.5	0.0	0.6	7.6	70.7
14	5	3.3	0.8	0.5	5.0	14.1	37.0	0.1	0.4	1.3	38.5
14	6	4.3	2.0	1.0	6.7	24.6	50.3	0.1	0.3	2.6	61.8
14	7	9.3	4.1	3.7	5.3	44.4	41.1	0.1	1.1	4.9	52.7
14	8	9.0	1.9	3.5	7.5	26.0	53.2	0.4	0.5	4.6	51.4
21	1	7.0	3.6	23.0	7.7	31.8	41.0	0.5	0.6	3.9	55.4
21	2	10.3	6.4	11.8	9.2	26.9	43.2	1.1	1.3	5.0	48.8
21	3	4.1	4.3	2.5	12.8	27.6	40.4	0.6	0.4	2.5	60.6
21	4	3.9	7.8	5.0	6.7	28.0	39.9	0.8	1.5	2.6	66.7
21	5	14.9	5.0	3.3	7.8	46.5	31.6	0.5	2.3	8.5	57.1
21	6	9.6	2.6	8.8	3.1	33.2	35.4	0.8	0.5	4.8	49.4
21	7	8.8	1.3	5.6	5.8	16.4	45.1	0.9	1.0	6.0	68.6
21	8	6.8	0.9	10.1	15.0	22.5	53.5	0.6	1.0	4.3	63.0
42	1	11.5	1.8	0.6	1.0	17.6	45.5	1.9	2.9	5.3	45.7
42	2	14.5	1.7	6.5	2.1	47.8	47.5	3.3	9.3	9.4	64.7
42	3	10.3	0.6	0.1	0.0	11.7	37.2	2.1	2.3	5.4	52.4
42	4	37.5	2.5	2.2	0.0	34.4	32.6	5.9	4.1	15.1	40.3
42	5	25.5	2.7	5.0	2.8	42.5	50.0	8.0	8.9	9.5	37.3
42	6	44.4	5.1	4.4	0.7	51.5	33.3	8.8	18.3	17.1	38.6

Table S2. List of antibodies

Antibody	Application	Host Animal	Dilution	Distributor	Cat. No., RRID
**Anti-CD4**	IHC-P	rabbit, monoclonal	1:1000	Abcam, Cambridge, UK	#ab183685, RRID: AB_2686917
**Anti-LYVE1**	IHC-P	goat, polyclonal	1:200	R&D Systems, Minneapolis, MN, USA	AF2125-SP, RRID: AB_2297188
**Anti-Foxp3**	IHC-P	rat, monoclonal	1:100	Thermo Fisher Scientific, Waltham, MA, USA	# 14-5773-80, RRID: AB_467575
**Anti-CD11c**	IHC-P	rabbit, polyclonal	1:100	Synaptic Systems, Göttingen, Germany	# HS-375003, RRID:
**Anti-Ly6G**	IHC-F	rat, monoclonal	1:400	Biolegend, San Diego, CA, USA	# 127601, RRID: AB_1089179
**Anti-F4/80**	IHC-F	rabbit, monoclonal	1:500	Cell Signaling Technology, Davers, MA, USA	# 70076, RRID: AB_2799771
**Anti-IL-33**	IHC-P, F	goat, polyclonal	1:200	R&D Systems, Minneapolis, MN, USA	# AF3626, RRID: 884269
**Anti-Keratin5**	IHC-P	rabbit, polyclonal	1:300	Biolegend, San Diego, CA, USA	# 905503, RRID: AB_2734679
**Anti-αSMA**	IHC-P	rabbit, monoclonal	1:200	Abcam, Cambridge, UK	# ab32575, RRID: AB_722538
**Anti-vimentin**	IHC-P	rabbit, monoclonal	1:200	Abcam, Cambridge, UK	#ab92547, RRID: AB_10562134
**Anti-HSP47**	IHC-P	rabbit, monoclonal	1:300	Abcam, Cambridge, UK	# ab109117, RRID: AB_10888995

**Anti-S100A4**	IHC-P	rabbit, monoclonal	1:2000	Abcam, Cambridge, UK	# ab197896, RRID: AB_2728774
**Anti-CD8**	IHC-P	rat, monoclonal	1:80	Thermo Fisher Scientific, Waltham, MA, USA	# 14-0808-82, RRID: AB_2572861
**Anti-CD45r**	IHC-P	rat, monoclonal	1:100	Gene Tex, Los Angeles, CA, USA	# GTX53152,
**Alexa Fluor 568-anti-Goat IgG**	IHC-P, F	donkey, polyclonal	1:400	Thermo Fisher Scientific, Waltham, MA, USA	#A-11057, RRID: AB_2534104
**Alexa Fluor 568-anti-Rabbit IgG**	IHC-P, F	donkey, polyclonal	1:400	Thermo Fisher Scientific, Waltham, MA, USA	#A-10042, RRID: AB_2534017
**Alexa Fluor 488-anti-Rabbit IgG**	IHC-P	donkey, polyclonal	1:400	Thermo Fisher Scientific, Waltham, MA, USA	#A-21206, RRID: AB_2535792
**Alexa Fluor 488-anti-Rat IgG**	IHC-P	donkey, polyclonal	1:400	Thermo Fisher Scientific, Waltham, MA, USA	#A-21208, RRID: AB_2535794
**Alexa Fluor 568-anti-Rat IgG**	IHC-F	donkey, polyclonal	1:400	Thermo Fisher Scientific, Waltham, MA, USA	#A78946 RRID: AB_2910653

IHC-P: Immunohistochemistry-Paraffin, IHC-F: Immunohistochemistry-Frozen
